# Objective to identify and verify the regulatory mechanism of DTNBP1 as a prognostic marker for hepatocellular carcinoma

**DOI:** 10.1038/s41598-021-04055-4

**Published:** 2022-01-07

**Authors:** Xianyi Cheng, Dezhi Li, Tiangyang Qi, Jia Sun, Tao Zhou, Wei V. Zheng

**Affiliations:** 1 Intervention and Cell Therapy Center, Peking University Shenzhen Hospital, Shenzhen, Guangdong People’s Republic of China; 2 Ascentawits Pharmaceuticals, Ltd., Biomedical Innovation Industrial Park, No. 14 Jinhui Road, Jinsha Community, Kengzi Street, Pingshan District, Shenzhen, Guangdong People’s Republic of China; 3 ShenZhen Beike Biotechnology Research Institute, Shenzhen, 518057 People’s Republic of China

**Keywords:** Oncogenes, Tumour biomarkers

## Abstract

Although the overall survival of hepatocellular carcinoma (HCC) patients has been significantly improved, prognostic clinical evaluation remains a substantial problem owing to the heterogeneity and complexity of tumor. A reliable and accurate predictive biomarker may assist physicians in better monitoring of patient treatment outcomes and follow the overall survival of patients. Accumulating evidence has revealed that DTNBP1 plays functional roles in cancer prognosis. Therefore, the expression and function of DTNBP1in HCC was systematically investigated in our study. The expression and prognostic value of DTNBP1 were investigated using the data from Cancer Genome Atlas (TCGA) database, Gene Expression Omnibus (GEO) cohorts and clinical samples. A series of cellular function assays were performed to elucidate the effect of DTNBP1 on cellular proliferation, apoptosis and metastasis. Kyoto Encyclopaedia of Genes and Genomes (KEGG) pathway enrichment and Protein–protein interaction (PPI) network construction were performed to screen the genes with highest interaction scores with DTNBP1. Finally, the underlying mechanism was also analyzed using Gene Set Enrichment Analysis (GSEA) and confirmed using RT-qPCR and western blotting. DTNBP1 was upregulated in many types of cancers, especially in HCC. The DTNBP1 expression levels is associated with clinicopathologic variables and patient survival status. The differential expression of DTNBP1 could be used to determine the risk stratification of patients with HCC. DTNBP1 deficiency inhibited cell proliferation and metastasis, but promoted cell apoptosis. Mechanistically, DTNBP1 regulated the cell cycle progression through affecting the expression of cell cycle-related genes such as CDC25A, CCNE1, CDK2, CDC20, CDC25B, CCNB1, and CDK1. DTNBP1, which regulates the cell cycle progression, may be used as a prognostic marker for HCC.

## Introduction

Globally, hepatocellular carcinoma (HCC) is the second leading cause of cancer-related deaths, thus necessitating development of more advanced treatment options and better treatment monitoring^1^. However, due to the combination of adverse factors with a range of different biological and clinical behaviours and the increased resistance to anti-HCC drugs, existing targeted drugs have shown unsatisfactory efficacy^2^. There is a lack of novel biomarkers for the evaluation of the clinical treatment of malignancies in HCC^3^. The lack of specific biomarkers for tumor stages or cancer subtypes represents a key gap during HCC therapy. Thus, it is necessary to develop an independent prognostic biomarker at the molecular level to determine liver cancer prognosis, especially considering the genomic and host factors that drive the progression. Investigation of factors can enhance our understanding of liver cancer biology, enable the development of enhanced screening strategies, and improve patient prognosis.

Dysbindin-1 (dystrobrevin binding protein-1, DTNBP1) is widely identified as a schizophrenia susceptibility gene^4, 5^. DTNBP1 expression level is significantly reduced in the brain of schizophrenia patients^6^. DTNBP1 expression disrupted both glutamatergic and gamma-aminobutyric acidergic transmission in the cerebral cortex^7^. Loss of DTNBP1 expression may result in development of deficient presynaptic vesicle transmission in the central nervous system^8^. The DTNBP1 mutation affected the GluN2B-GluN2A switch at the synapse in a brain-region-specific fashion via pY1472-GluN2B, Fyn, and PLCγ during NMDA receptor dysfunction^9^. Moreover, DTNBP1 is involved in auditory and visual processing^10^. DISC1 functionally interacts with the fly homolog of DTNBP1 via direct protein–protein interaction in developing synapses^11^. Additionally, DNA methylation levels of DTNBP1 promoter were considered for the diagnosis of schizophrenic individuals^12^, which partially suggested that DTNBP1 might be regulated in the liver via methylation processes.Accumulating evidence suggests that DTNBP1 functions in cancer, suggesting that it may regulate and interact with all three D-type cyclins (cyclin D1, D2, and D3) and affect cell cycle progression^4^. Additionally, DTNBP1 is a potential biomarker for PDAC detected via the serum mass spectrometry (MS) proteomic profiling assay^13^. In brain tumors, single-nucleotide polymorphisms within the gene *DTNBP1* were significantly associated with attention, executive functioning, and memory scores in patients with brain tumors^14^. However, the biological function of DTNBP1 in HCC remains unclear, and whether DTNBP1 can serve as a predictor for prognosis need to be determined.

In this study, for the first time, we found that the DTNBP1 levels in HCC tissues were significantly higher than those in normal liver tissues according to the Cancer Genome Atlas (TCGA) database^15^, Gene Expression Omnibus (GEO) cohorts, and immunohistochemical staining of HCC microarray. Higher DTNBP1 level indicated shorter overall survival (OS). We formed a nomogram of OS based on DTNBP1 and TNM stage to estimate the risk. DTNBP1 deficiency inhibited cell proliferation and metastasis, but promoted cell apoptosis. Mechanistically, DTNBP1 regulated the cell cycle progression through affecting the expression of cell cycle-related genes such as CDC25A, CCNE1, CDK2, CDC20, CDC25B, CCNB1, and CDK1. The present study may reveal a novel biomarker for the prognosis assessment in HCC.

## Methods

### Data mining from TCGA and GEO databases

Gene expression profiles (https://portal.gdc.cancer.gov/) of 416 LICH patients in the cohort TCGA-LICH were investigated. The clinical data and survival status information of 419 LIHC patients were downloaded from the UCSC Xena website (https://tcga.xenahubs.net with cohort: TCGA-LICH). Among them, 339 patients with whole clinical and survival data were subjected to further analysis. The raw counts of RNA were log-normalized and analyzed using the edge R package (3.30.3). The threshold was log2FC |(fold change)|> 0.5 p-value < 0.001. The DTNBP1 gene expression profile was analyzed using tools: GEPIA (http://gepia.cancer-pku.cn/). The GEO dataset GSE45436 (NT41, HCC62), GSE46408 (NT6, HCC6), GSE64041 (normal 5, tumor&non-tumor120), GSE76427 (NT52, HCC115), GSE101685 (normal 8, HCC24), GSE112790 (normal 15, HCC 183) were downloaded from the GEO website to evaluate the difference in GNPNAT1 expression in normal tissues compared to that tumor tissues. The Human Protein Atlas (HPA) database (https://www.proteinatlas.org/) was used to investigate the DTNBP1 protein spatial expression in normal tissues and tumor tissues.

### Construction of a predictive nomogram

Independent prognostic measures were performed to construct a nomogram using the software R: A Language and Environment for Statistical Computing 2020 (R Foundation for Statistical Computing, Vienna, Austria. URL: https://www.R-project.org/) with the Regression Modelling Strategies package. The calibration plot was generated for estimating the calibration value of the nomogram.

### Immunohistochemistry (IHC)

The tissue microarray, which contained 75 samples obtained from HCC tissues and 75 samples obtained from corresponding adjacent normal tissues, was purchased from Shanghai Outdo Biotech Company (Shanghai, China). The tissue slides were deparaffinized, treated with 3% H_2_O_2_, subjected to antigen retrieval by autoclaving in the presence of 10 mM citric sodium (pH 6.0) for 30 min to unmask antigens, rinsed in PBS, and then incubated with the primary antibodies against DTNBP1 at 4 °C overnight, followed by incubation with biotinylated secondary antibody for 1 h at room temperature. Signal amplification and detection were performed using DAB (MXB Biotechnologies, Fuzhou, China). German semi-quantitative scoring system was used to score staining intensity, according to the intensity of the nucleic, cytoplasmic and membrane staining (no staining = 0, weak staining = 1, moderate staining = 2, strong staining = 3). The DTNBP1 expression level was semi-quantified using the formula: level = intensity score × positive rate × 100.

### Ethics approval and consent to participate

The tissue array was formed from tissues of consenting donors. The use of this array was approved by Ethics Committee of Shanghai Outdo Biotech Company (SHYJS-CP-1904006). The datasets accessed were publicly available and anonymized, and therefore formal ethical approval and informed consent was not required. All experiments were performed in accordance with relevant guidelines and regulations.

### Cell lines and cell culture

Normal liver cell line THLE-2 and HCC cell lines (Hep3B, HepG2, Huh7, and PLC/PRF/5) were obtained from Xiamen Immocell Biotechnology Co., Ltd (Xiamen, China). All cells were cultured in DMEM (Gibco, Detroit, MI, USA) containing 10% fetal bovine serum (FBS, Gibco), 100 U/mL penicillin (Gibco), 100 U/mL streptomycin (Gibco), and 2 mM l-glutamine (Gibco) at 37 °C in a 5% CO_2_ incubator.

### shRNA transfection

The shRNA-1, shRNA-2, and shRNA-3 were synthesized by and purchased from GenScript Biotech (Nanjing, China) based on the sequence shown in Supplementary Table 1. The medium was replaced with FBS-free DMEM and then the cells were transfected with shRNA using Lipofectamine 2000 (Invitrogen; Thermo Fisher Scientific, Inc.) at 37 °C. After a 12-h incubation, the medium was replaced with DMEM containing 10% FBS. The cells were harvested for further experiments after corresponding time. Cells were lysed 50 h post-transfection, and DTNBP1 expression was analyzed using RT-qPCR and western blotting. Each experiment was conducted in triplicate.

### Reverse transcription-quantitative PCR (RT-qPCR)

Total RNA was extracted from cells 50 h post-transfection using the RNA Isolater Total RNA Extraction Reagent (Vazyme, Nanjing, Jiangsu, China) and then was subjected to reverse transcription using Superscript III Reverse Transcriptase (Invitrogen, Thermo Fisher Scientific, Inc.) at 47 °C for 50 min. The qPCR assay was conducted using the ChamQ SYBR qPCR Master Mix (Vazyme Biotech Co., Ltd.). The thermocycling conditions were as follows: 98 °C for 30 s, followed by 40 cycles of 98 °C for 5 s, 60 °C for 15 s. Each reaction was performed in triplicate, and expression levels were normalized to those of 18S ribosomal RNA. The qPCR primers are listed in Supplementary Table 1.

### Western blotting

Protein was extracted from the cells using mammalian protein extraction reagent (Sangon Biotech Co., Ltd, Shanghai, China) 50 h post-transfection. Equal amounts of protein (20 μg per lane), as estimated by using a bicinchoninic acid (BCA) protein assay kit (Abcam, Shanghai, China), were loaded into wells of 10% denaturing SDS-PAGE gels and transferred onto a PVDF membrane (Millipore, MA, USA). The blots obtained were probed with a monoclonal antibody, followed by addition of the secondary HRP-conjugated anti-mouse/rabbit antibody. Detailed information on the antibodies is listed in Supplementary Table 2. After washing, the blots were subjected to chemiluminescence, and relative optical densities were analyzed using the image processing software (Image J). Relative protein content was calculated by dividing the optical density of the target band using the optical density of the GAPDH band obtained. Bands on the film from different projects were respectively cropped. Therefore, the original images of blots were not full-length.

### Assessment of cell viability

These cells were seeded in 96-well plates at a density of 4 × 10^4^ cells per well. Cell proliferation was assessed using an MTT kit (Cat: 40201ES72, Yeasen, Shanghai, China) at each time point or drug concentration for 60 min at 37 °C. Each experiment was performed in sextuple.

### Detection of apoptosis

Twenty-two hours after performing cell treatment experiments, cells were seeded into 6-well plates at a density of 1 × 10^5^ cells per well. The cells were then collected and stained using Annexin V-fluorescein isothiocyanate (FITC) and 30 mg/mL PI (Cat: A211-02, Vazyme, Nanjing, China), and apoptosis was subsequently determined using a flow cytometer NovoCyte 1300 (ACEA, San Diego, CA, USA).

### EdU incorporation assay

Twenty-two hours after performing cell treatment experiments, cells were labeled with EdU for 4 h and then fixed to stain the cells using the EdU staining kit BeyoClick™ EdU-488 (Cat: C0071S, Beyotime, Shanghai, China). The EdU-positive cells were then observed and photographed under a fluorescence microscope (MOTIC, Hongkong, China). The number of cells were counted using Image J 1.52v (NIH, Bethesda, MD, USA).

### Cell cycle analysis

Forty-eight hours after performing cell treatment experiments, cells were harvested and washed in PBS, followed by fixation in chilled 70% ethanol for 30 min at 4 °C. The cells were washed and treated with 20 µL ribonuclease (100 µg/mL). Addition of 200 µL PI (50 µg/mL stock solution) was performed to stain the cells, and cells were further analyzed by flow cytometry.

### Cell migration and invasion

Twenty-two hours after performing cell treatment experiments, cells were harvested and suspended with DMEM. Eighty microliters of cell suspensions containing 6 × 10^4^ cells were placed above the filter membrane of Transwell plates (Cat: 3422, Corning, Corning, NY, USA) and incubate for 24 h at 37 °C and 5% CO_2_. For invasion assay, additional extracellular matrix materials (Cat: 356234, BD Biosciences, Sparks, MD, USA) were added on the Transwell membrane. For staining, 600 μL 70% ethanol was used to fix the cells and 0.2% crystal violet was applied for staining^16^. The staining results were detected under a light microscope (MOTIC) and the number of cells was counted using Image J 1.52v.

### Functional enrichment analysis and protein–protein interaction (PPI) network construction

Select DTNBP1 co-expression genes from the CBioPortal database (https://www.cbioportal.org/) with the Spearman's correlation p-value < 0.001. These genes were taken to DAVID (https://david.ncifcrf.gov/) for enrichment analysis. The Kyoto encyclopedia of genes and genomes (KEGG) pathway categories were identified with p values < 0.05 and enrichment scores > 2 were considered statistically significant and were plotted using the R package ggplot2^17–19^. Genes contributing to the leading-edge subset within the gene set were defined using the Leading Edge analysis (https://www.gsea-msigdb.org/gsea/doc/GSEAUserGuideFrame.html). Then the genes with |Spearman's Correlation coefficient value|> 0.4 were analyzed using the STRING website (https://string-db.org/) to examine the relationship among these genes, followed by generation of the network map using the Cytospace software.

### Gene set enrichment analysis (GSEA)

GSEA software (v4.0.3) was used to explore the mechanisms of DTNBP1 expression on the progression of HCC. CP (canonical pathways): KEGG gene sets (n = 186) was obtained from MSigDB database V7.2. The Nominal p-value < 0.05 was considered to be significantly enriched.

### DTNBP1 overexpression and transfection

cDNA of DTNBP1 was amplified using the primers shown in Supplementary Table 1. And was cloned into the vector pCDH-CMV-MCS-T2A-BSD (Antihela, Xiamen, Fujian, China). Huh7 cells were seeded into 6-well at the density of 2 × 10^6^ per well. The cells in “Vector” and “DTNBP1 OE” groups were transfected with empty vector (4 μg/well) and pCDH-DTNBP1 plasmid (4 μg/well) using Lipofectamine 2000, respectively. Twenty-four hours after transfection, the cells groups were harvested for analyse of cellular function as above. All experiments were performed in triple.

### Statistical analysis

All statistical analyses were performed using SPSS Statistics version 19.0 (IBM Corp., Armonk, NY, USA) and GraphPad Prism version 8.0.2 (GraphPad Software, Inc.). Mann–Whitney test was performed for nonparametric data between two groups. Wilcoxon matched-pairs signed rank test was used to compare matched samples for nonparametric data. Kruskal–Wallis one-way ANOVA followed by Dunn’s multiple comparison test were performed for nonparametric data among multiple groups. The chi-square test was used to analyze the correlation between DTNBP1 expression levels and clinical features of the patients. Survival curves were calculated using the Kaplan–Meier method, and the significance was determined by the log-rank test. The independent indicators related to OS were identified using Cox proportional hazards model, and the hazard ratios (HR) with 95% confidence intervals (CI) were also calculated. Nomograms were constructed based on the results of Cox multivariate analyses in terms of OS. By combined evaluation of the C-index and calibration, the performance of the established nomograms was effectively measured. One way ANOVA followed by Tukey’s post-hoc test was used for multiple comparisons among three groups of parametric data. The level of statistical significance was set at *P* < 0.05.

## Results

### DTNBP1 expression is upregulated in HCC patients

**Figure 1:**
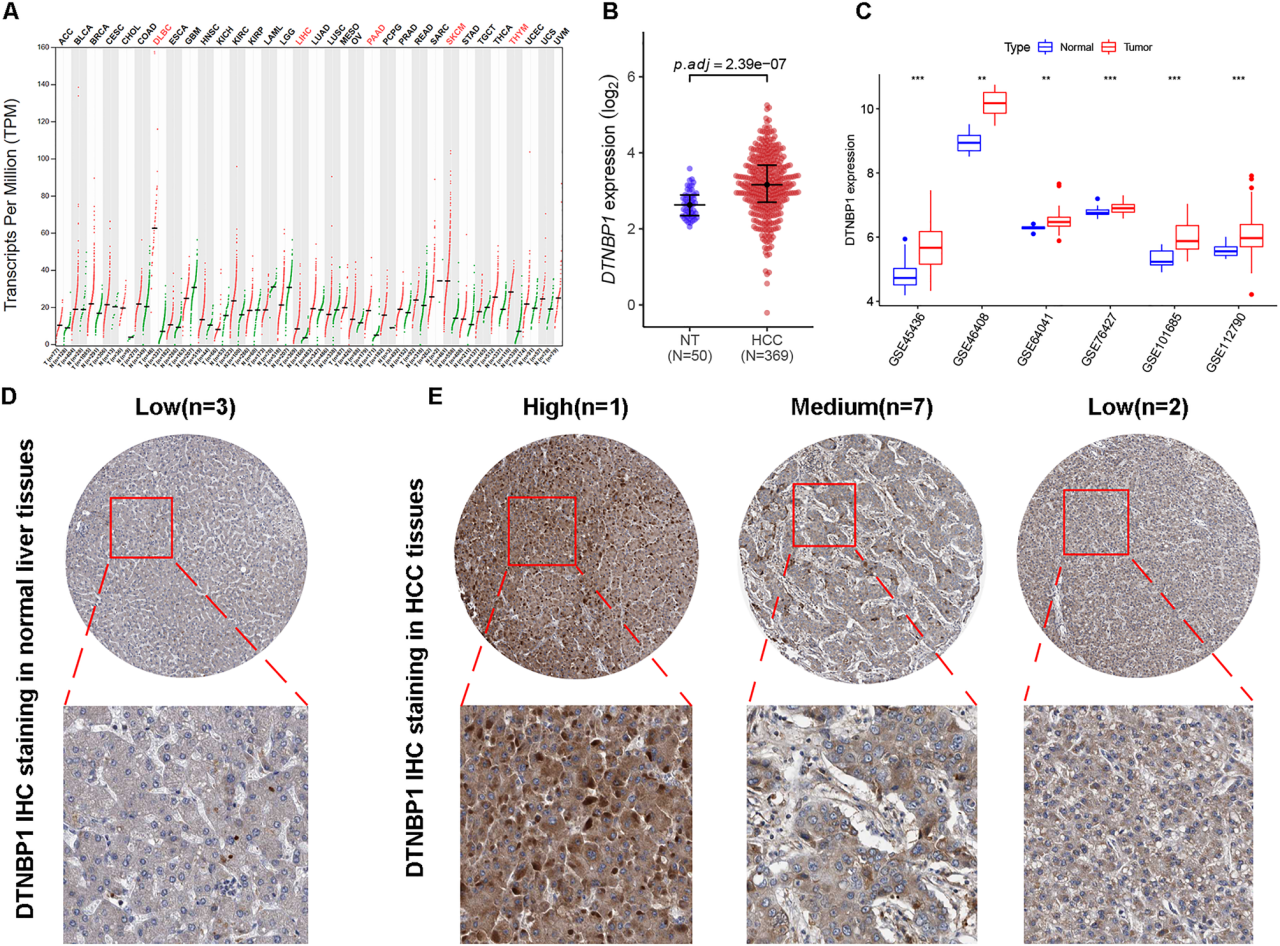
Expression of DTNBP1 was examined using the public databases. (**A**) Pan-cancer analysis of DTNBP1 mRNA expression (TPM) using the GePia database. ACC, Adrenocortical cancer; BLCA, Bladder Urothelial Carcinoma; BRCA, Breast invasive carcinoma; CESC, Cervical squamous cell carcinoma and endocervical adenocarcinoma; CHOL, Cholangio carcinoma; COAD, Colon adenocarcinoma; DLBC, Lymphoid Neoplasm Diffuse Large B-cell Lymphoma; ESCA, Esophageal carcinoma; GBM, Glioblastoma multiforme; HNSC, Head and Neck squamous cell carcinoma; KICH, Kidney Chromophobe; KIRC, Kidney renal clear cell carcinoma; KIRP, Kidney renal papillary cell carcinoma; LAML, Acute Myeloid Leukemia; LGG, Brain Lower Grade Glioma; LIHC, Liver hepatocellular carcinoma; LUAD, Lung adenocarcinoma; LUSC, Lung squamous cell carcinoma; MESO, Mesothelioma; OV, Ovarian serous cystadenocarcinoma; PAAD, Pancreatic adenocarcinoma; PCPG, Pheochromocytoma and Paraganglioma; PRAD, Prostate adenocarcinoma; READ, Rectum adenocarcinoma; SARC, Sarcoma; SKCM, Skin Cutaneous Melanoma; STAD, Stomach adenocarcinoma; TGCT, Testicular Germ Cell Tumors; THCA, Thyroid carcinoma; THYM, Thymoma; UCEC, Uterine Corpus Endometrial Carcinoma; UCS, Uterine Carcinosarcoma; UVM, Uveal Melanoma; (**B**) Scatter plot displayed the differential expressions of DTNBP1 between the normal tissues (NT, n = 50) and hepatocellular carcinoma (HCC, n = 369) in TCGA dataset. Mann–Whitney test was used for statistical analysis. (**C**) Box plot illustrating the differential expression of DTNBP1 between the normal tissues and tumor tissues in the 6 GEO datasets. Mann–Whitney test: **P < 0.01; ***P < 0.001. (**D**) Representative image of immunohistochemistry (IHC) demonstrates DTNBP1 expression in normal liver tissues from The Human Protein Atlas (HPA) database. (**E**) Representative IHC image demonstrates DTNBP1 expression in tumor tissues from HPA database.

**Figure 2:**
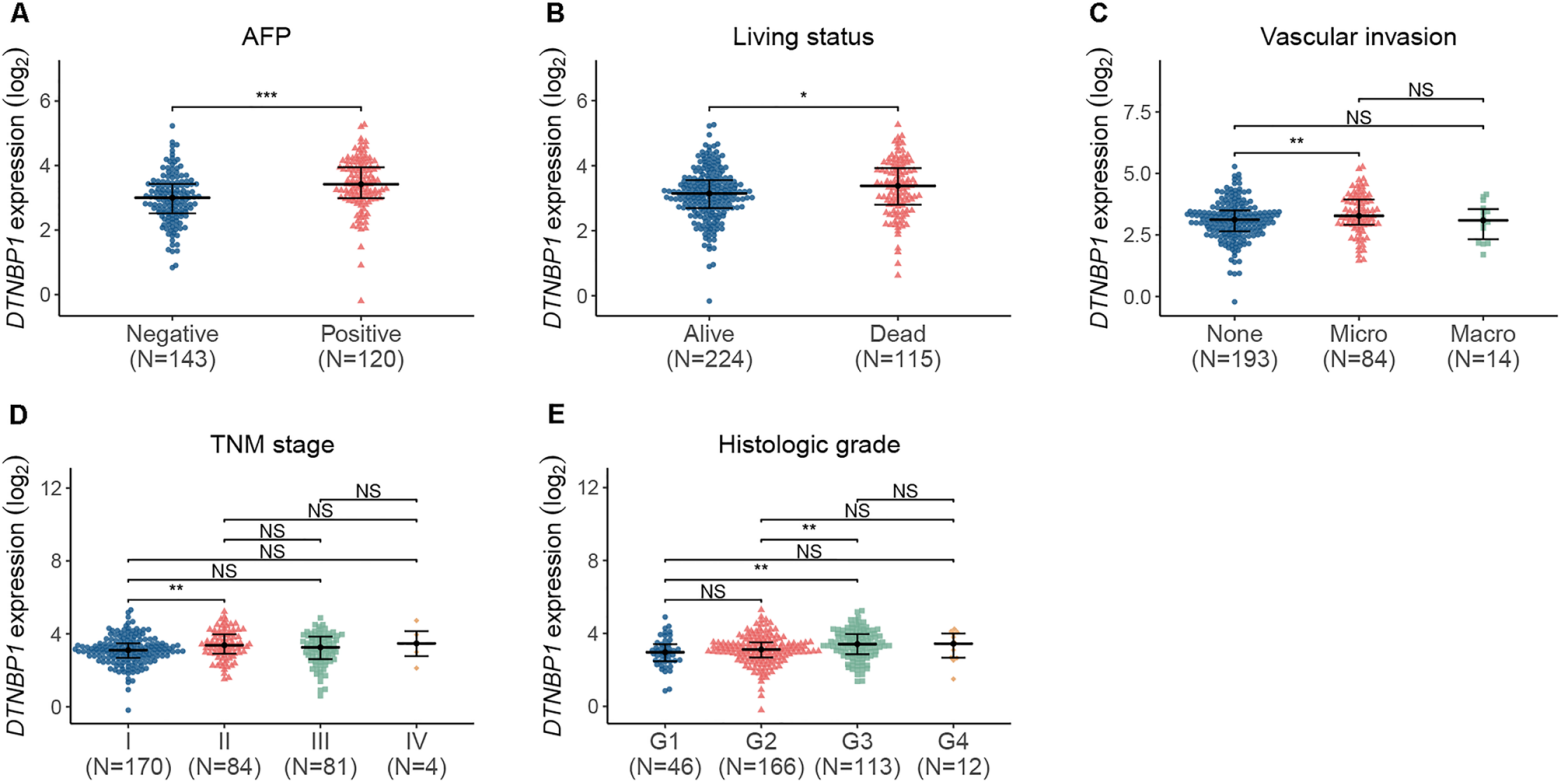
Scatter plot showing the differential expression analysis of DTNBP1 in TCGA patients, as divided by (**A**) AFP-negative (n = 143) and AFP-positive (n = 120) statuses. (**B**) Living status: alive (n = 224) and dead (n = 115). (**C**) Vascular invasion status: none (n = 193), microinvasion (micro, n = 84), and microinvasion (macro, n = 14). (**D**) TNM stage: Stage I (I, n = 170), Stage II (II, N = 84), Stage III (III, n = 81), and Stage IV (IV, n = 4). (**E**) Histologic grade: Grade 1 (G1, n = 46), Grade 2 (G2, n = 116), Grade 3 (G3, n = 113), and Grade 4 (G4, n = 12). Mann–Whitney test was performed for statistical analysis of (**A**) and (**B**). Kruskal–Wallis one-way ANOVA followed by Dunn’s multiple comparison test were used for statistical analysis of (**C**), (**D**), and (**E**). NS, no significance; *p < 0.05; **p < 0.01; ***p < 0.005.

First, we analyzed the differentially expressed genes in TCGA dataset by using Edge R and found that DTNBP1 expression was upregulated in the hepatocellular carcinomas (Fig. S1). Furthermore, we investigated the DTNBP1 gene expression profile in 33 cancer types both in both normal and paired tumor samples. We found that the mRNA expression of DTNBP1, as determined via TPM analysis, was significantly upregulated in TCGA dataset: DLBC, LIHC, PAAD, SKCM, THYM (Fig. 1A). To further validate the differential expression pattern of DTNBP1 in LIHC, normal tissues and tumor tissues were compared in terms of their DTNBP1 mRNA levels. As shown in Fig. 1B, the mRNA levels of DTNBP1 were higher in tumor tissues than those in the normal controls. Additionally, five GEO datasets were used as the testing datasets. Consistent with the findings obtained from TCGA analysis, DTNBP1 mRNA level in tumor tissues was also significantly higher than that in normal liver tissues (Fig. 1C). Furthermore, we investigated the protein expression of DTNBP1 used the Human Protein Atlas (HPA) database. As shown in Fig. 1D,E, The DTNBP1 was mainly expressed in the hepatocytic cells. The positive signal was distributed in the cytoplasm. The HCC tissues displayed higher DTNBP1 level compared to normal liver tissues. In addition, we analyzed the differential expression of DTNBP1 in the subgroup divided by the clinical features. As shown in Fig. 2A, alpha-fetoprotein (AFP)-positive patients had higher DTNBP1 mRNA level than AFP-negative patients. DTNBP1 mRNA level was lower in patients who were alive than in dead patients (Fig. 2B). Notably, higher mRNA level of DTNBP1 was observed in patients with micro vascular invasion than in non-vascular patients (Fig. 2C). Moreover, higher DTNBP1 mRNA level was observed in TNM stage II and G3 (Fig. 2D,E) than in their corresponding controls. The correlation between clinicopathological variables and DTNBP1 expression level in HCC was summarized in Table 1. Statistical analysis revealed that DTNBP1 level was significantly correlated with age, gender, histologic grade, ATP, and living status. There was no significant correlation between DTNBP1 level and other clinicopathological variables including Family history of cancer, TNM stage, Ishak score, Child–Pugh grade, vascular invasion, residual tumor, and disease status.

**Table 1:** Correlation between clinicopathological variables and DTNBP1 expression in HCC.

Clinicopathological variables	Total	DTNBP1 expression		p-value ^a^
		High	Low	
	(N = 339)	(N = 110)	(N = 229)	
**Age (year)**				
< 65	208 (61.4%)	78 (70.9%)	130 (56.8%)	***0.017***
≥ 65	131 (38.6%)	32 (29.1%)	99 (43.2%)	
**Gender**				
Male	231 (68.1%)	66 (60.0%)	165 (72.1%)	***0.035***
Female	108 (31.9%)	44 (40.0%)	64 (27.9%)	
**Family history of cancer**				
No	196 (57.8%)	69 (62.7%)	127 (55.5%)	0.447
Yes	98 (28.9%)	28 (25.5%)	70 (30.6%)	
Unknown	45 (13.3%)	13 (11.8%)	32 (14.0%)	
**TNM stage**				
I	170 (50.1%)	46 (41.8%)	124 (54.1%)	0.186
II	84 (24.8%)	32 (29.1%)	52 (22.7%)	
III	81 (23.9%)	30 (27.3%)	51 (22.3%)	
IV	4 (1.2%)	2 (1.8%)	2 (0.9%)	
**Histologic grade**				
G1–G2	212 (62.5%)	53 (48.2%)	159 (69.4%)	** < *0.001***
G3–G4	125 (36.9%)	56 (50.9%)	69 (30.1%)	
Unknown	2 (0.6%)	1 (0.9%)	1 (0.4%)	
**Ishak score**				
0–4	124 (36.6%)	33 (30.0%)	91 (39.7%)	0.197
5–6	74 (21.8%)	25 (22.7%)	49 (21.4%)	
Unknown	141 (41.6%)	52 (47.3%)	89 (38.9%)	
**Child–pugh grade**				
A	207 (61.1%)	63 (57.3%)	144 (62.9%)	0.128
B–C	21 (6.2%)	4 (3.6%)	17 (7.4%)	
Unknown	111 (32.7%)	43 (39.1%)	68 (29.7%)	
**Vascular invasion**				
None	193 (56.9%)	52 (47.3%)	141 (61.6%)	0.094
Micro	84 (24.8%)	33 (30.0%)	51 (22.3%)	
Macro	14 (4.1%)	5 (4.5%)	9 (3.9%)	
Unknown	48 (14.2%)	20 (18.2%)	28 (12.2%)	
**Alpha fetoprotein**				
Negative	143 (42.2%)	34 (30.9%)	109 (47.6%)	** < *0.001***
Positive	120 (35.4%)	54 (49.1%)	66 (28.8%)	
Unknown	76 (22.4%)	22 (20.0%)	54 (23.6%)	
**Residual tumor**				
R0	301 (88.8%)	97 (88.2%)	204 (89.1%)	0.777
R1-R2	12 (3.5%)	5 (4.5%)	7 (3.1%)	
Unknown	26 (7.7%)	8 (7.3%)	18 (7.9%)	
**Living status**				
Alive	224 (66.1%)	60 (54.5%)	164 (71.6%)	***0.003***
Dead	115 (33.9%)	50 (45.5%)	65 (28.4%)	
**Disease status**				
No	163 (48.1%)	54 (49.1%)	109 (47.6%)	0.967
Yes	132 (38.9%)	42 (38.2%)	90 (39.3%)	
Unknown	44 (13.0%)	14 (12.7%)	30 (13.1%)	
**Disease specific**				
No	266 (78.5%)	79 (71.8%)	187 (81.7%)	0.106
Yes	65 (19.2%)	27 (24.5%)	38 (16.6%)	
Unknown	8 (2.4%)	4 (3.6%)	4 (1.7%)	

^a^Chi-square test. Statistically significant p-value was given in bold italic.

**Figure 3:**
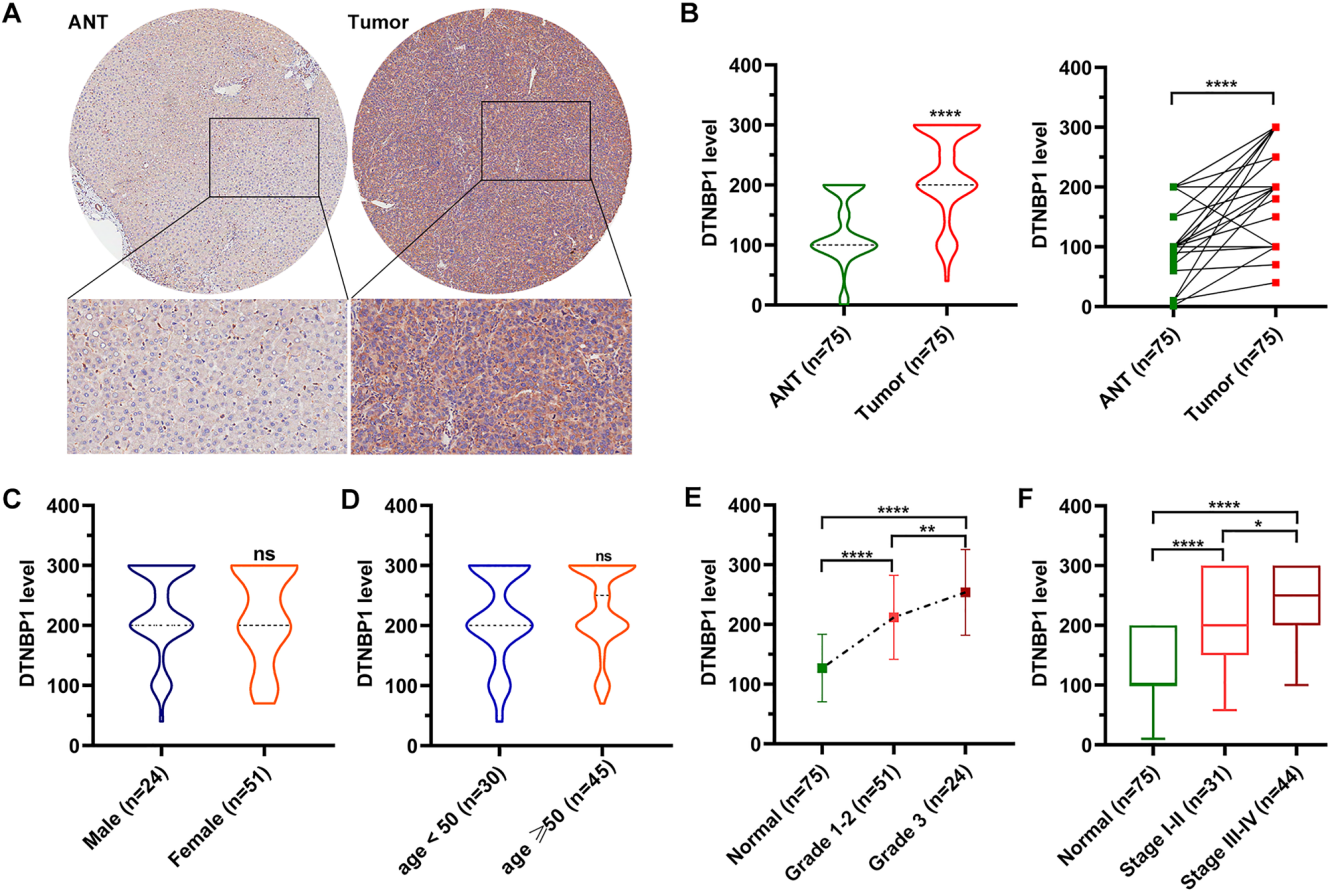
Validation of the clinical significance of DTNBP1 using HCC microarray. (**A**) Representative image of DTNBP1 IHC staining. (**B**) The DTNBP1 protein level was higher in tumor tissues, compared with ANT. Statistical analysis: left panel, Mann–Whitney test; right panel, Wilcoxon matched-pairs signed rank test. (C-D) Violin plot illustrating the differential expression of DTNBP1 in subgroups of patients divided by gender (**C**) and age (**D**). Mann–Whitney test was used for statistical analysis. (**E**) Plot illustrating the differential expression of DTNBP1 in subgroups of patients divided by historical stage. Kruskal–Wallis one-way ANOVA followed by Dunn’s multiple comparison test was used for statistical analysis. (**F**) Box plot illustrating the differential expression of DTNBP1 in subgroups of patients divided by TNM stage. The data are analyzed using Kruskal–Wallis one-way ANOVA test followed by Dunn’s multiple comparison test. ns, no significance; *p < 0.05; **p < 0.01; ****p < 0.0001.

To further validate the clinical value of DTNBP1 in clinical applications, we performed immunohistochemistry to stain DTNBP1 in commercial HCC microarray (Fig. 3A). The DTNBP1 expression levels were higher in tumor tissues than those in adjacent normal tissues (Fig. 3B). No significant differences were observed in clinical factors, such as age and sex (Fig. 3C,D), while DTNBP1 protein levels were significantly different in the subgroups when groups were divided by the histological stage and TNM stage (Fig. 3E,F).

Taken together, these findings demonstrated that DTNBP1 is overexpressed in HCC patients and is further increased as the patient’s progress moves to a more lethal stage, indicating that DTNBP1 may serve as an oncogenic factor determining HCC progression.

### Higher DTNBP1 level indicated shorter OS time of HCC patients

**Figure 4:**
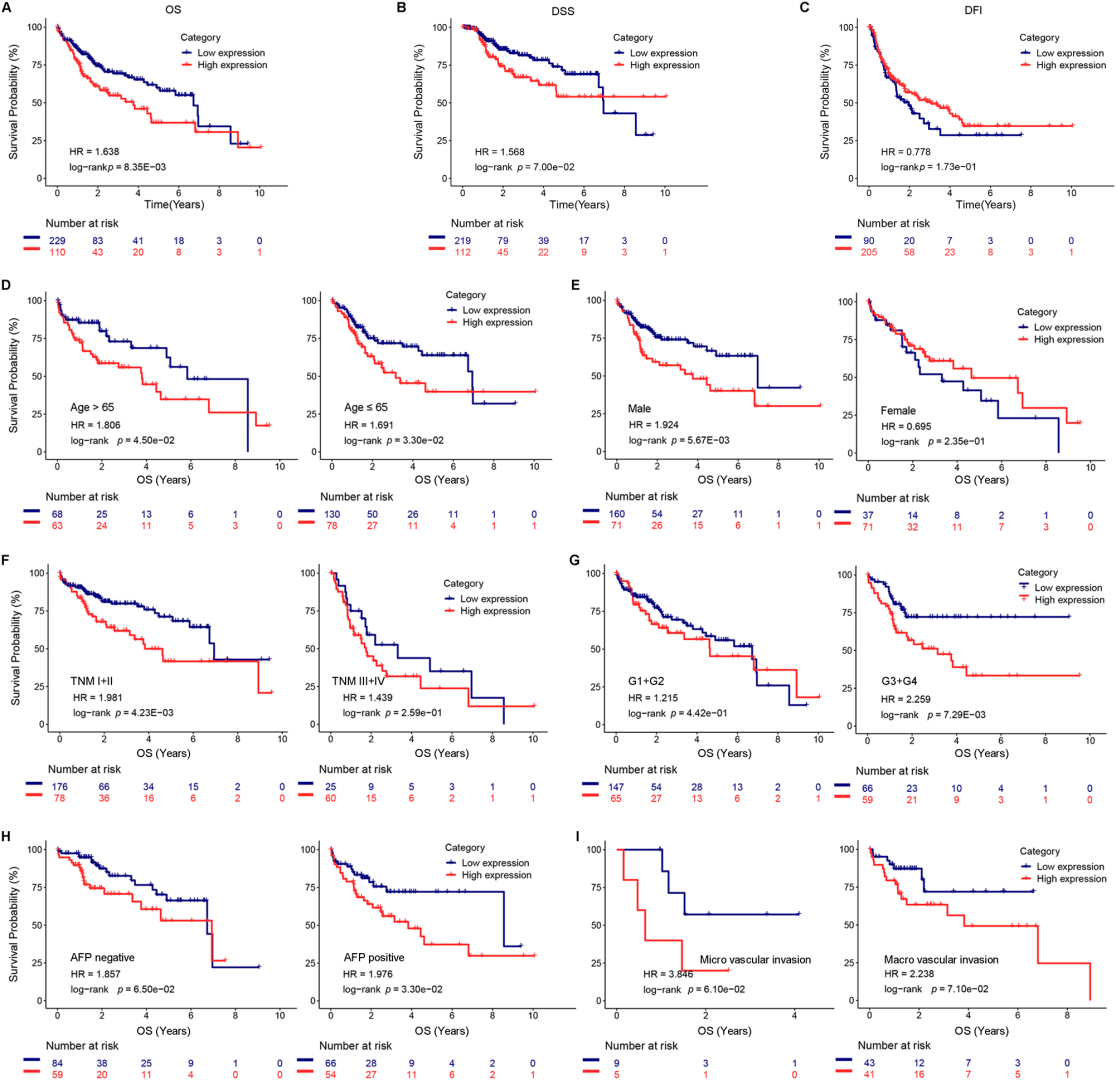
Higher DTNBP1 level indicates shorter overall survival (OS) time in HCC patients. (**A**) The relationship of DTNBP1 level with OS. (**B**) The association between DTNBP1 level and disease-specific survival (DSS). (**C**) The association of DTNBP1 level with disease-free interval (DFI). The association between DTNBP1 level and OS in HCC patients classified by age > 65 or age ≤ 65 (**D**), male or female (**E**), TNM stage I + II or TNM stage III + IV (**F**), G1 + G2 or G3 + G4 (**G**), alpha-fetoprotein (AFP)-positive or AFP-negative (**H**), micro or macro vascular invasion (**I**).

Additionally, we investigated the relationship of DTNBP1 level with OS, disease-specific survival (DSS), and disease-free interval (DFI). As shown in Fig. 4A, patients in the high DTNBP1 group had a shorter OS. However, there was no significant difference in DSS (Fig. 4B) or DFI (Fig. 4C) found between the high DTNBP1 and low DTNBP1 groups. Furthermore, we did more analyses on OS with other clinicopathological variables. Stratified analyses also illustrated that high DTNBP1 level served as a factor for poor prognosis, when patients were classified as age > 65 years (Fig. 4D, left, p = 0.0450), age ≤ 65 years (Fig. 4D, right, p = 0.0330), male (Fig. 4E, left, p = 0.0057), TNM stage I + II (Fig. 4F, left, p = 0.0042), G3 + G4 (Fig. 4G, right, p = 0.0073), and AFP positive (Fig. 4H, right, p = 0.0330). Notably, the DTNBP1 level failed to predict the OS of patients categorized as female (Fig. 4E, right, p = 0.2350), TNM stage III + IV (Fig. 4F, right, p = 0.2590), G1 + G2 (Fig. 4G, left, p = 0.4420), AFP negative (Fig. 4H, left, p = 0.6500), micro vascular invasion (Fig. 4I, left, p = 0.0610), and micro vascular invasion (Fig. 4I, right, p = 0.0710). In addition, using univariate and multivariate Cox analyses, DTNBP1 and TNM stage were observed to also function as significant independent predictors of OS (Table 2). Taken together, these findings suggested that DTNBP1 is a useful prognostic factor of HCC.

**Table 2:** Cox proportional hazards regression model analysis of overall survival.

Variables	Univariate analysis		Multivariate analysis	
	HR (95% CI)	P-value	HR (95% CI)	P-value
Age (≥ 65 vs. < 65)	1.23 (0.85,1.78)	0.273	–	–
Gender (female vs. male)	1.26 (0.87,1.84)	0.228	–	–
Family history of cancer (yes vs. no)	1.14 (0.76,1.69)	0.530	–	–
TNM stage (II vs. I)	1.42 (0.87,2.32)	***0.160***	1.08 (0.56,2.07)	0.818
TNM stage (III vs. I)	2.72 (1.78,4.15)	** < *0.001***	2 (1.15,3.5)	***0.015***
TNM stage (IV vs. I)	5.44 (1.68,17.63)	***0.005***	5.25 (1.55,17.74)	***0.008***
Histologic grade (G3–G4 vs. G1–G2)	1.14 (0.78,1.67)	0.489	–	–
Ishak score (5–6 vs. 0–4)	0.87 (0.5,1.5)	0.612	–	–
Child–Pugh grade (B–C vs. A)	1.66 (0.82,3.36)	0.159	1.5 (0.72,3.13)	0.281
Vascular invasion (micro vs. none)	1.16 (0.72,1.88)	0.539	0.88 (0.49,1.59)	0.677
Vascular invasion (macro vs. none)	2.52 (1.14,5.58)	***0.023***	1.84 (0.79,4.3)	0.156
Alpha fetoprotein (positive vs. negative)	1.45 (0.92,2.28)	***0.108***	1.18 (0.7,1.99)	0.533
Residual tumor (R1–R2 vs. R0)	1.17 (0.43,3.2)	0.754	–	–
DTNBP1 (high vs. low)	1.21 (0.97,1.51)	***0.089***	1.32 (1,1.72)	***0.046***

Statistically significant P value is given in bold italic.

CI: confidence interval. HR: hazard ratio.

### Development and validation of a DTNBP1-predicting nomogram

**Figure 5:**
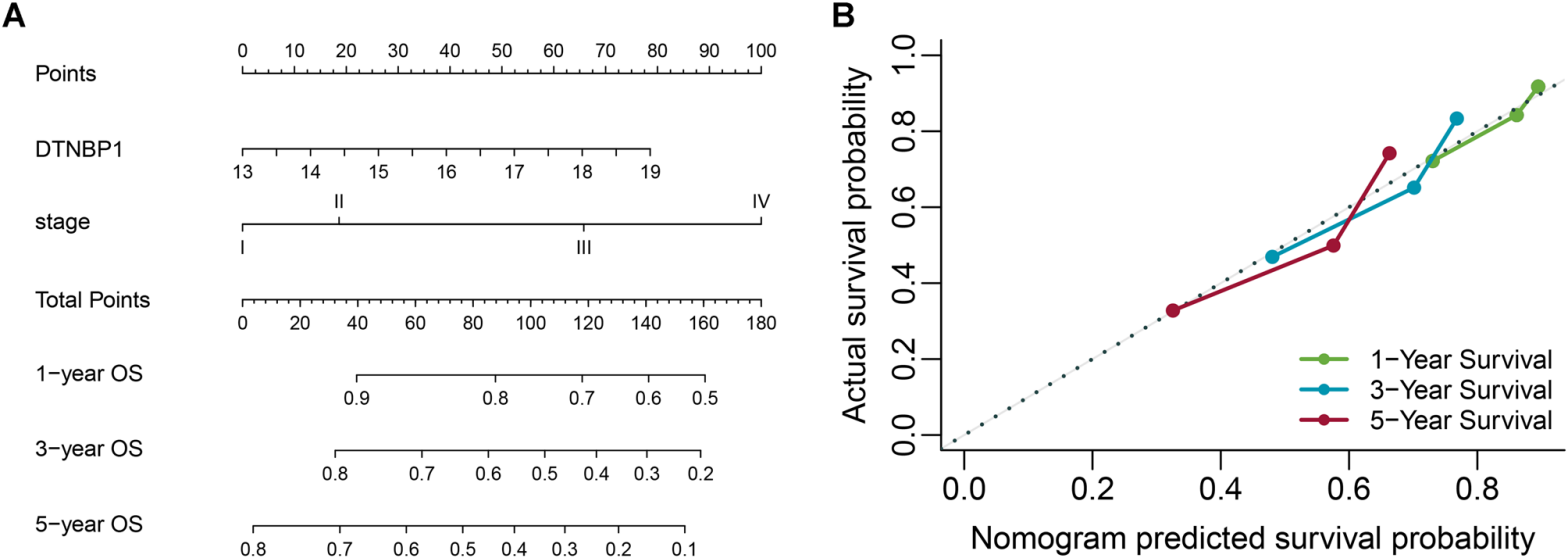
Nomogram for estimation of DTNBP1 risk and predictive performance. (**A**) Nomogram of the overall survival (OS) as an endpoint. Individual value is plotted on each variable axis, and a line is drawn upward to determine the number of points received for each variable value. The summed numbers are located on the Total Points axis, and a line represents the survival axes to determine the likelihood of 1-, 3-, or 5-year survival. (**B**) Calibration curve of the OS Nomogram prediction; probability of overall survival is plotted on the x-axis; actual overall survival is plotted on the y-axis.

The two independent predictors, DTNBP1 and TNM stage, were used to form a DTNBP1 expression level-related risk estimation nomogram (Fig. 5). The nomogram demonstrated excellent accuracy in estimating the risk of DTNBP1 in OS, with a C-index of 0.63 (95% CI 0.60–0.66) (Fig. 5A). Additionally, calibration plots graphically showed adequate agreement on the presence of DTNBP1 between the risk estimation by the nomogram and histopathologic confirmation (Fig. 5B).

### Downregulation of DTNBP1 expression reduces cell proliferation and increases apoptosis

**Figure 6:**
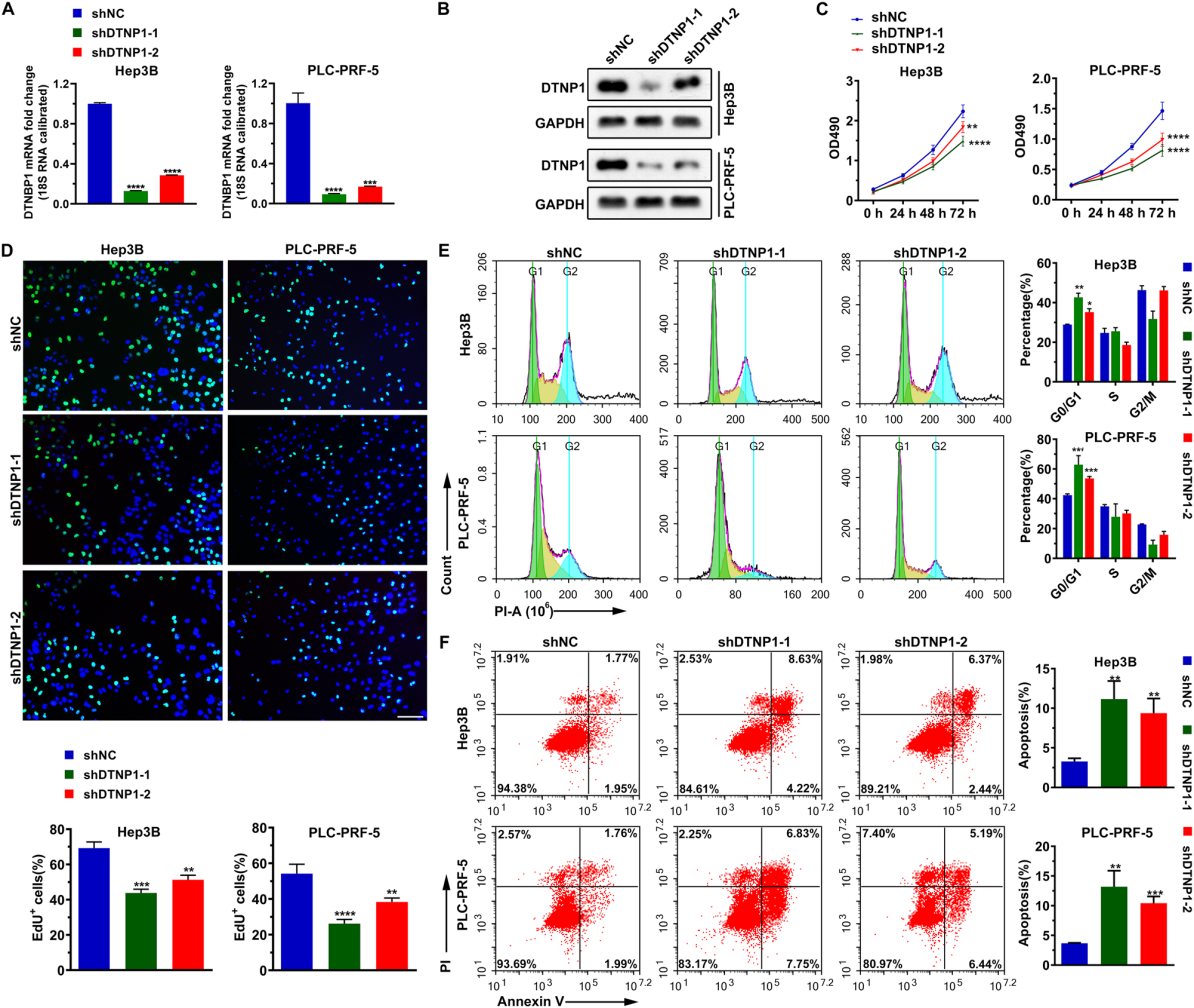
DTNBP1 expression levels influence the cell status. (**A**) The mRNA expression levels of DTNBP1 were analyzed using q-PCR when the cells were transfected with DTNBP1 shRNAs (shRNA1, shRNA2). The relative mRNA levels were normalized to 18S rRNA expression levels. (**B**) The protein expression levels of DTNBP1 were analyzed by western blotting when the cells were transfected with DTNBP1 shRNAs. GAPDH was used as the loading control. The bolts were cropped from different gels with the same exposure time. (**C**) The cell viability of the cells was measured using the MTT assay. (**D**) Representative images of EdU incorporation, cells stained with EdU and DAPI, and results were quantitatively illustrated using the bar plot. Bar: 10 μm. (**E**) Representative FACS images of cell cycle analysis based on PI staining and its quantification illustrated using the bar plot. (**F**) Representative FACS images of cell apoptosis analysis based on Annexin V/PI staining and its quantification illustrated using the bar plot. One way ANOVA followed by Tukey’s post-hoc test: *p < 0.05; **p  < 0.01; ***p  < 0.001; ****p  < 0.0001.

To verify the functional roles of DTNBP1 in cell functions, we first compared its expression level between normal liver cell lines and HCC cell lines. As shown in Fig. S2, both mRNA and protein levels of DTNBP1 in the HCC cell lines except for Huh7 were significantly higher than those in the normal liver cell line THLE-2. Hep3B and PLC-PRF-5, which had the highest levels of DTNBP1, were chosen for further experiments. We used short hairpin RNAs (shRNAs) against DTNBP1 to knock down DTNBP1 in Hep3B and PLC-PRF-5. Indeed, we observed that the DTNBP1 shRNAs (shDTNBP1-1 and shDTNBP1-2) reduced both mRNA and protein levels of DTNBP1 in Hep3B and PLC-PRF-5 (Fig. 6A,B). Knockdown of DTNBP1 could inhibit cell viability, as evidenced by the results of the MTT assay (Fig. 6C). Furthermore, the EdU staining assay showed that the percentage of cells in replication (the EdU-positive cells) significantly decreased when DTNBP1 was knocked down (Fig. 6D). As shown in Fig. 6E, the DTNBP1-knockdown group showed that the cell cycle was arrested in the G_0_/G_1_ phase, which implied that the cell cycle progression was inhibited. Furthermore, the percentage of apoptosis was significantly increased in the DTNBP1-knockdown group (Fig. 6F). Taken together, our results demonstrated that reduced levels of DTNBP1 inhibit cell proliferation and increases apoptosis in HCC cell lines.

### Downregulation of DTNBP1 expression inhibits the metastasis of HCC cells

**Figure 7:**
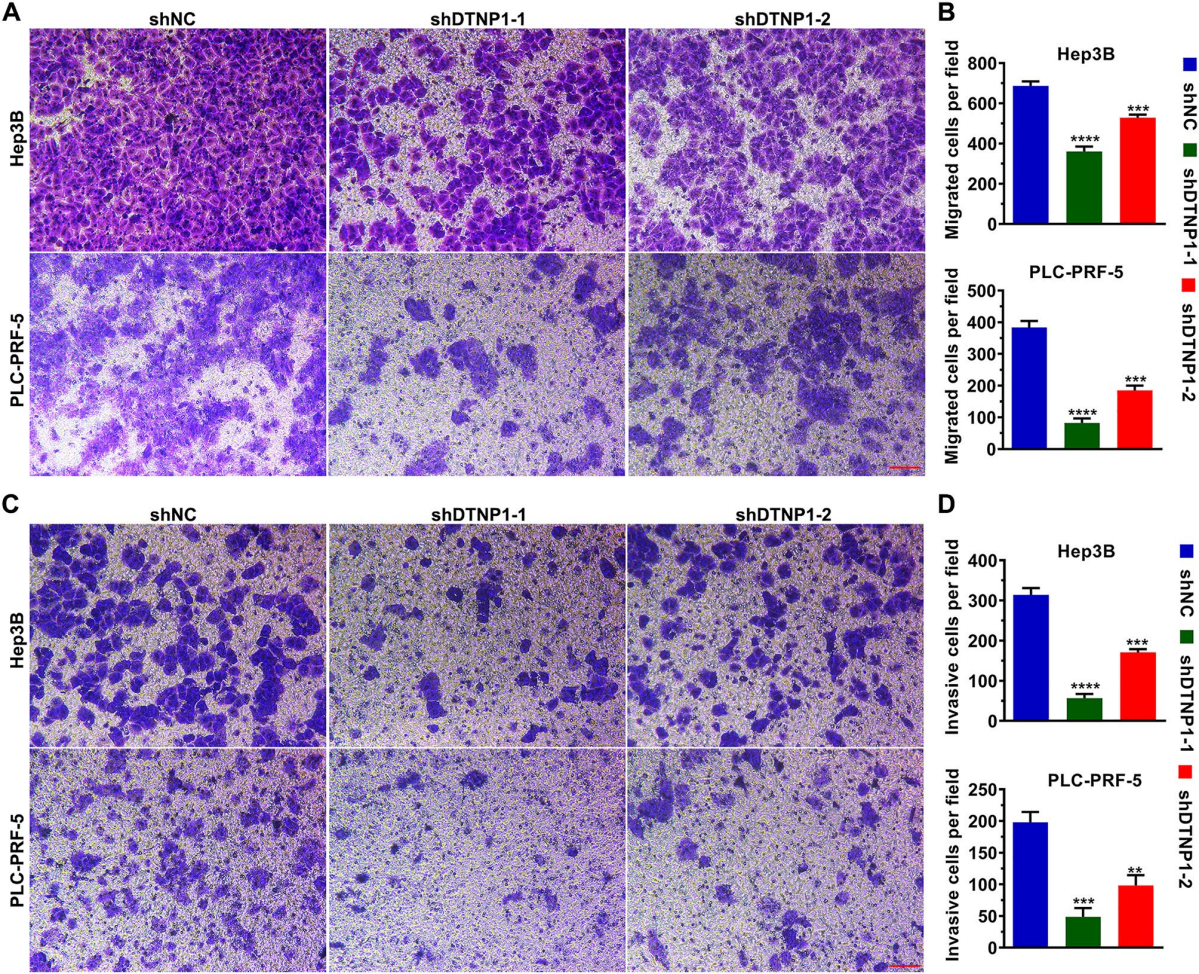
Downregulation of DTNBP1 expression inhibits the abilities of migration and invasion. (**A**) Representative images of Transwell migration assay when cells were transfected with a DTNBP1 knockdown plasmid or its control vector. (**B**) Quantification of the migrated cells per field presented in (**A**). (**C**) Representative images of Transwell invasion when cells were transfected with DTNBP1 knock-down plasmid or its control vector. (**D**) Quantification of the migrated cells per field presented in (C). One way ANOVA followed by Tukey’s post-hoc test: **p < 0.01; ***p  < 0.001; ****p  < 0.0001.

Results from the Transwell migration assays demonstrate that the DTNBP1-knockdown group exhibited significantly decreased cell migration compared to the cells transfected with the empty vector (Fig. 7A,B). Additionally, results from the Matrigel invasion assays demonstrate that DTNBP1 knockdown significantly reduced the number of invasive cells (Fig. 7C,D). Taken together, downregulation of DTNBP1 expression suppresses cell metastasis.

### DTNBP1 regulatory network analysis

**Figure 8:**
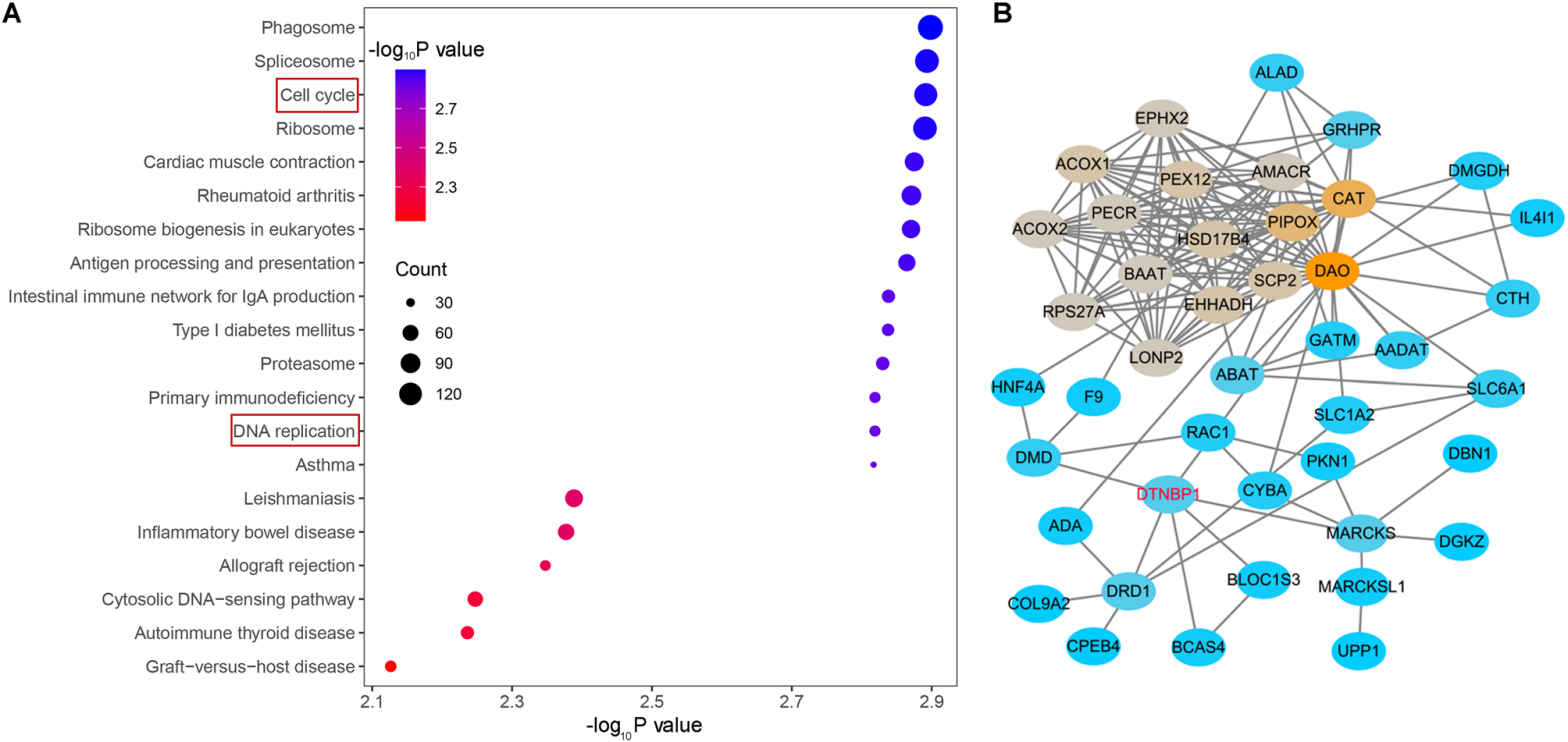
Functional enrichment analysis and protein–protein interaction (PPI) network construction. (**A**) Bubble blot illustrating the top 20 significantly enriched KEGG pathways. (**B**) PPI network map showing 43 genes concerning the highest interaction scores with DTNBP1.

To investigate the biological classification of DTNBP1 in HCC, we conducted functional enrichment analysis of genes that co‐expressed with DTNBP1. The top 20 pathways concerning the significantly enriched KEGG pathways showed that DTNBP1 was significantly related with the processes of cell cycle and DNA replication (Fig. 8A), suggesting that DTNBP1 potentially facilitates the growth of HCC cells. The PPI network was illustrated in Fig. 8B, showing the 43 genes which have the highest interaction scores with DTNBP1.

### DTNBP1 regulates the expression levels of cell cycle-related proteins

**Figure 9:**
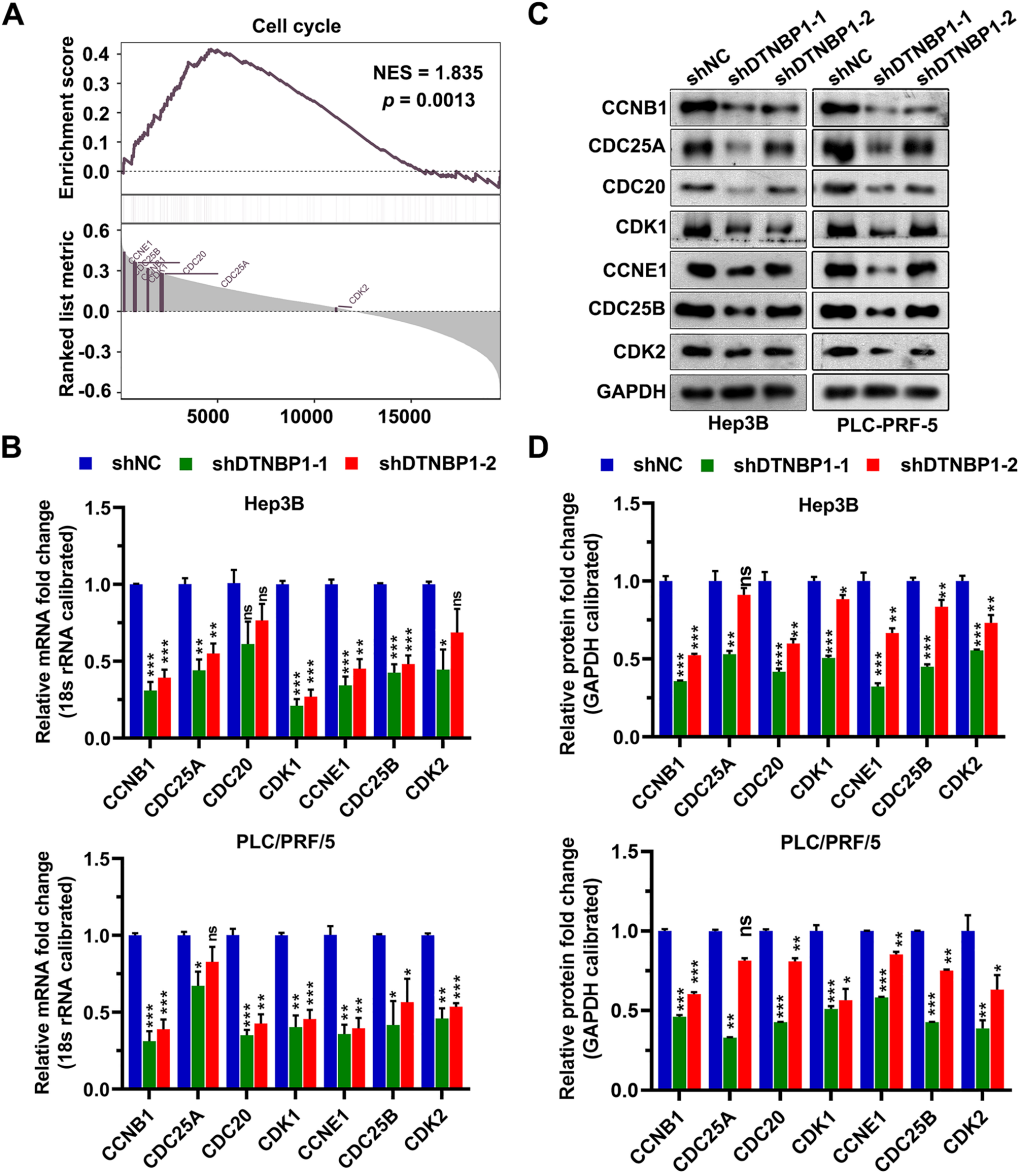
Downregulation of DTNBP1 expression decreased the expression of cell cycle related proteins. (**A**) GSEA analysis of the top 20 co-expressed genes of DTNBP1. (**B**) The mRNA levels of CCNB1, CDC25A, CDC20, CDK1, CCNE1, CDC25B, and CDK2 were detected using RT-qPCR. (**C**) The protein levels of CCNB1, CDC25A, CDC20, CDK1, CCNE1, CDC25B, and CDK2 were detected using western blotting. The bolts were cropped from different gels with different exposure times. (D) Quantitation of band intensity with values normalized to GAPDH. One way ANOVA followed by Tukey’s post-hoc test: ns, no significance; *p < 0.05; **p < 0.01; ***p < 0.001.

To further investigate the potential signaling pathways involved in DTNBP1-inhibitory effects in cancer cells, we performed GSEA to explore the biological function of DTNBP1 upregulation in HCC, and “cell cycle” pathway was significantly enriched (Fig. 9A). The core genes in the both pathways were listed in the Supplementary Table 3. We further investigated the effect of DTNBP1 expression on the mRNA levels of cell cycle-related genes including *CCNB1, CDC25A, CDC20, CDK1, CCNE1, CDC25B,* and *CDK2*. As shown in Fig. 9B, DTNBP1 knockdown significantly reduced the mRNA expression levels of these genes. In consistent with the mRNA levels, the protein levels of these genes were also significantly reduced after DTNBP1 deficiency with the exception of CDC25A in group shDTNBP1-2 for PLC-PRF-5 cells (Fig. 9C,D). These data indicated that DTNBP1 regulates cell cycle progression.

### Overexpression of DTNBP1 promotes cell cycle progression

**Figure 10:**
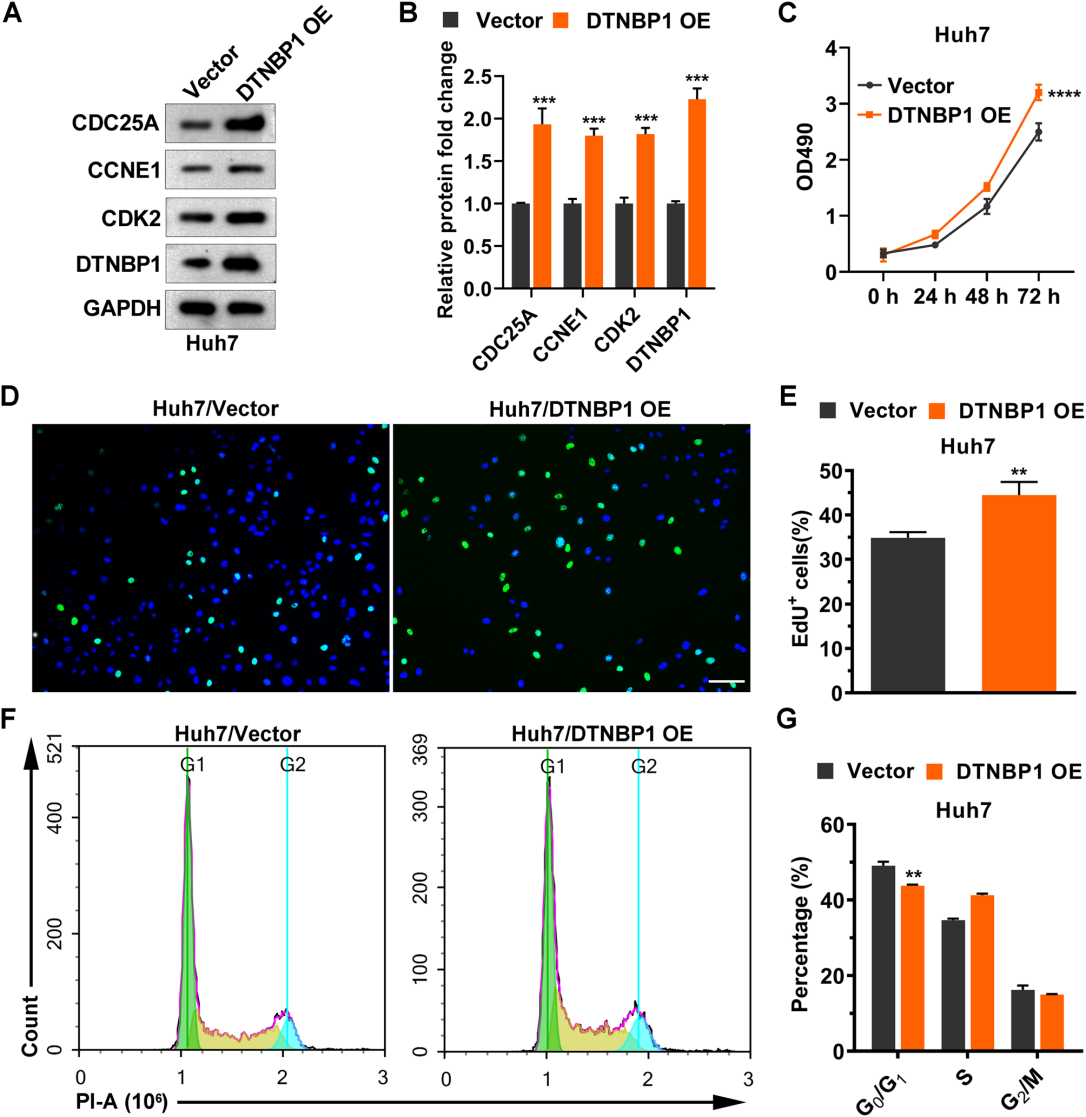
DTNBP1 overexpression promotes cell cycle progression. (**A**) The protein levels of CDC25A, CCNB1, CDK2, and DTNBP1 were detected using western blotting. The bolts were cropped from different gels with different exposure times. (**D**) Quantitation of band intensity with values normalized to GAPDH. (**C**) MTT assay. (**D**) Representative images of EdU incorporation. Bar: 10 μm. (**E**) Quantification of (**D**) using the bar plot. (**F**) Representative FACS images of cell cycle analysis. (**G**) Quantification of (**F**) using the bar plot. Unpaired Student’s t test: **p < 0.01; ***p  < 0.001; ****p  < 0.0001.

To further confirm the regulation of DTNBP1 on cell cycle and growth, we overexpressed DTNBP1 in Huh7 cells. As shown in Fig. 10A,B, DTNBP1 overexpression increased the protein levels of CDC25A, CCNE1, and CDK2. Moreover, high level of DTNBP1 promoted the cell proliferation (Fig. 10C), DNA replication (Fig. 10D,E), and passing through G_0_/G_1_ phase (Fig. 10F,G). These data suggested that DTNBP1 acts as an oncogene by promoting cell cycle progression.

## Discussion

An independent prognostic biomarker is helpful for the evaluation of the clinical treatment of malignancies in HCC. In this study, a novel and efficient prognostic biomarker, DTNBP1, was investigated in HCC patients. DTNBP1 was significantly upregulated in HCC tissues and demonstrated a high correlation with clinical features and OS of patients. DTNBP1 and TNM stage are both independent predictors for patient prognosis. It seems that TNM stage outperforms DTNBP1 expression in the OS prediction. However, we should use both of them to form a nomogram of OS to estimate the risk. The nomogram was credible and available. The association between high DTNBP1 expression in HCC and unfavourable characteristics (high AFP value, high TNM stage, poor OS, etc.) makes us wonder whether a high DTNBP1 expression might simply represent an epiphenomenon of a poorly differentiated and aggressive histotype instead of playing a primary causative role. Thus, we investigated the effect of DTNBP1 on the cellular function including cellular proliferation, apoptosis, and metastasis after DTNBP1 knockdown in HCC cell lines Hep3B and PLC/PRF/5. The in vitro experiments confirmed that DTNBP1 acts as an oncogene. Moreover, KEGG enrichment analysis revealed that the enriched pathways were related to DNA replication and cell cycle. Our results of EdU and cell cycle assays showed that DTNBP1 knockdown inhibited the cell replication and induced the arrest at G_0_/G_1_ phase, which was consistent with the conclusion of KEGG enrichment analysis. As we know, during the progression of the cell cycle, cyclin E1 (CCNE1) functions as a regulatory subunit of CDK2, which is required for G_1_/S progression^20^. CDK2 is dephosphorylated to be activated by CDC25A^21^. Thus, downregulation of CDC25A, cyclin E1 (CCNE1), and CDK2 can induce G_0_/G_1_ arrest^22–24^. CDC20, CDC25B, cyclin B1 (CCNB1), and CDK1 are critical for cells entering mitosis^25–27^. However, our results showed that the reduction of forementioned molecules induced by DTNBP1-deficiency only caused G_0_/G_1_ arrest without G_2_/M arrest. The reason may be that the cells were synchronized by starvation and the G_0_/G_1_ phase was the first stage of interphase. Our findings may provide a potential biomarker for malignancy and survival predictions in HCC patients.

Our analysis of bioinformatics revealed that DTNBP1 is upregulated in several other cancer types, indicating that the specificity of DTNBP1 as a diagnostic marker for HCC may be not enough. However, DTNBP1 may be used as a universal predictor for OS in different kinds of cancer, which is need to be further confirmed.

We did a univariate Cox analysis on individual variable and out of those which became significant (p < 0.20) should be subjected to multivariate Cox analysis (Table 2). The threshold of univariate Cox analysis was set at p value < 0.20 rather than 0.05 to prevent from missing the significant independent risk factors^28^.

There are several new biomarkers for HCC have been reported in the recent years. Lai et al. demonstrated that GRPEL2 is highly expressed in the HCC and plays well in predicting OS in patients with the same gender, age, pathological grade or clinical stage^29^. CDT1 and RMI2 are also reported to be overexpressed in HCC and act as independent predictors for OS in HCC patient^30, 31^. We suggest using TNM stage, DTNBP1, GRPEL2,CDT1, and RMI2 to form a predicting nomogram, which should be more accurate and credible.

It should be noted that there are also several limitations for this study. For example, we need to uncover the mechanism of DTNBP1 on apoptosis and metastasis in the next work. Moreover, in vivo experiments should be performed to validate our conclusion.

## Conclusions

In summary, we identified a novel biomarker DTNBP1 in HCC. Furthermore, DTNBP1 expression may reveal relevant cell cycle progression in HCC patients and may also help the prognosis assessment in HCC.

## Supplementary Information

Supplementary Figure 1.

Supplementary Figure 2.

Supplementary Table 1.

Supplementary Table 2.

Supplementary Table 3.

Supplementary Information 6.

## Data Availability

The data that support the findings of this study are available from the corresponding author upon reasonable request.
