# Supplementary figures and images for: Objective to identify and verify the regulatory mechanism of DTNBP1 as a prognostic marker for hepatocellular carcinoma
Source: Sci Rep. 2022 Jan 7;12:211. doi: 10.1038/s41598-021-04055-4 (PMC8742032; doi:10.1038/s41598-021-04055-4)

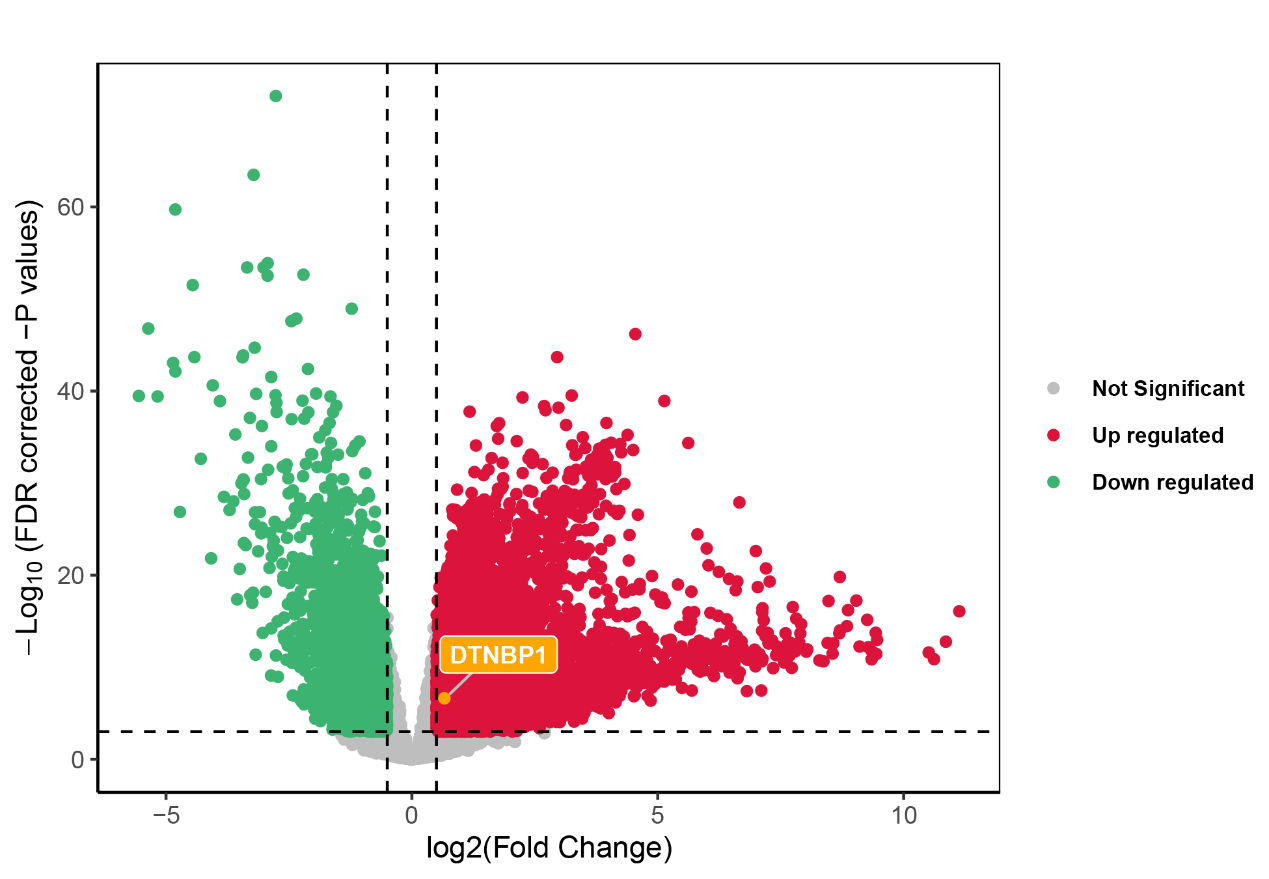


**Fig. S1** DTNBP1 was up-regulated in the hepatocellular carcinomas

Supplement: Supplementary file 1 — Supplementary Figure 1. [file 41598_2021_4055_MOESM1_ESM.docx]
