# Supplementary material for: Objective to identify and verify the regulatory mechanism of DTNBP1 as a prognostic marker for hepatocellular carcinoma
Source: Sci Rep. 2022 Jan 7;12:211. doi: 10.1038/s41598-021-04055-4 (PMC8742032; doi:10.1038/s41598-021-04055-4)
Supplement: Supplementary file 2 — Supplementary Figure 2. [file 41598_2021_4055_MOESM2_ESM.docx]

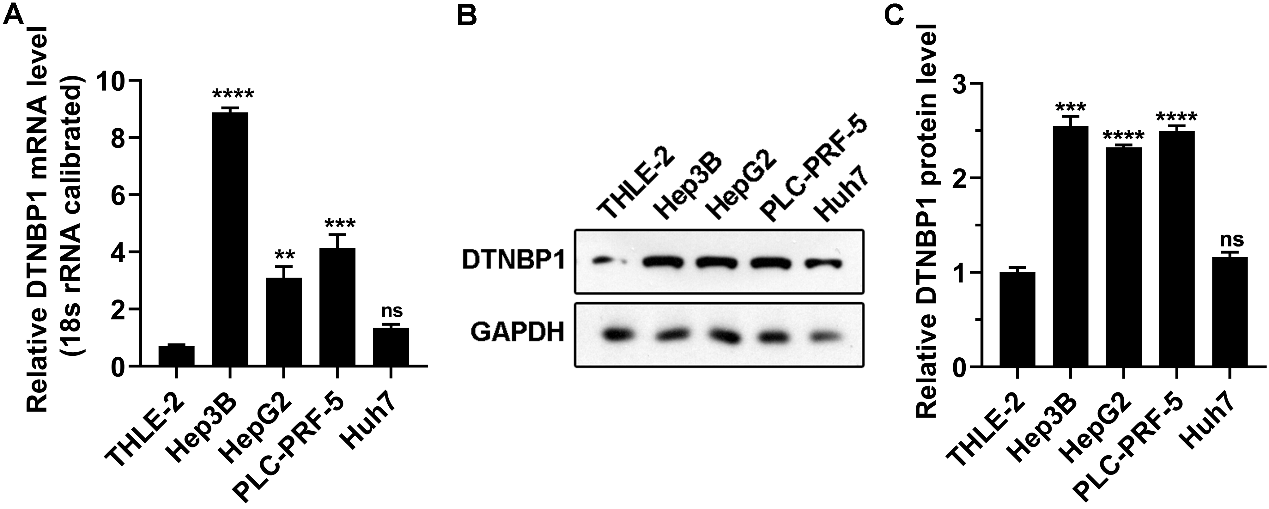


**Fig. S2** The expression level of DTNBP1 in cell lines. (A) RT-qPCR analysis showing the mRNA level of DTNBP1 in cell lines. 18s rRNA was used as an internal control. (B) Analysis of western blotting showing the protein level of DTNBP1 in cell lines. (C) Quantification of DTNBP1 band intensity, with values normalized to GAPDH. One way ANOVA followed by Tukey’s post-hoc test: ns, no significance; **, P<0.01; ***, P<0.001; ****, P<0.0001.
