# Supplementary material for: Objective to identify and verify the regulatory mechanism of DTNBP1 as a prognostic marker for hepatocellular carcinoma
Source: Sci Rep. 2022 Jan 7;12:211. doi: 10.1038/s41598-021-04055-4 (PMC8742032; doi:10.1038/s41598-021-04055-4)
Supplement: Supplementary file 3 — Supplementary Table 1. [file 41598_2021_4055_MOESM3_ESM.docx]

**Supplemental table 1. primers for plasmid construction and qPCR**

| **Name** | | **Sequence（5′-3′）** |
| --- | --- | --- |
| shNC | F | CCGGTTCTCCGAACGTGTCACGTTTCTCGAGAAACGTGACACGTTCGGAGAATTTTT |
| shDTNBP1-1 | R  F | AATTAAAAATTCTCCGAACGTGTCACGTTTCTCGAGAAACGTGACACGTTCGGAGAA  CCGGGCTGAAGACTTTAAGTGACAACTCGAGTTGTCACTTAAAGT CTTCAGCTTTTT |
| shDTNBP1-2 | R  F | AATTAAAAAGCTGAAGACTTTAAGTGACAACTCGAGTTGTCACTT AAAGTCTTCAGC  CCGGGGTGAGGACAGCGACTCTTAACTCGAGTTAAGAGTCGCTG TCCTCACCTTTTT |
| DTNBP1 OE | R  F  R | AATTAAAAAGGTGAGGACAGCGACTCTTAACTCGAGTTAAGAGTCGCTGTCCTCACC  TTGAGGAAGCCTTCCAGCAGGA  TCAGGTCCATCTGCTCCAGCAT |
| 18S | QF | AGGCGCGCAAATTACCCAATCC |
|  | QR | GCCCTCCAATTGTTCCTCGTTAAG |
| CCNB1 | QF | GACCTGTGTCAGGCTTTCTCTG |
|  | QR | GGTATTTTGGTCTGACTGCTTGC |
| CDC25A | QF | TCTGGACAGCTCCTCTCGTCAT |
|  | QR | ACTTCCAGGTGGAGACTCCTCT |
| CDC20 | QF | CGGAAGACCTGCCGTTACATTC |
|  | QR | CAGAGCTTGCACTCCACAGGTA |
| CDK1 | QF | GGAAACCAGGAAGCCTAGCATC |
|  | QR | GGATGATTCAGTGCCATTTTGCC |
| CCNE1 | QF | TGTGTCCTGGATGTTGACTGCC |
|  | QR | CTCTATGTCGCACCACTGATACC |
| CDC25B | QF | AGAACCTCCTGGACAGTGACCA |
|  | QR | GCTGAACTTGCCCGTCAATAGG |
| TGFB1 | QF | TACCTGAACCCGTGTTGCTCTC |
|  | QR | GTTGCTGAGGTATCGCCAGGAA |
| DTNBP1 | QF | TAATCGCAGACTTAGAAT |
|  | QR | CAAGTTCCTTCCTCTTAT |

F: forward primer. R: reverse primer. QF: forward primer for qPCR. QR: reverse primer for qPCR.
