# Supplementary material for: Objective to identify and verify the regulatory mechanism of DTNBP1 as a prognostic marker for hepatocellular carcinoma
Source: Sci Rep. 2022 Jan 7;12:211. doi: 10.1038/s41598-021-04055-4 (PMC8742032; doi:10.1038/s41598-021-04055-4)
Supplement: Supplementary file 4 — Supplementary Table 2. [file 41598_2021_4055_MOESM4_ESM.docx]

**Supplementary table 2. Antibodies for western blotting**

| **Antibody** | **Supplier** | **Catalog number** | **Dilution** |
| --- | --- | --- | --- |
| Anti-CCNB1 | Proteintech | 55004-1-AP | 1:2000 |
| Anti-CDC25A | Proteintech | 55031-1-AP | 1:1500 |
| Anti-CDC20 | Proteintech | 10252-1-AP | 1:500 |
| Anti-CDK1 | Proteintech | 19532-1-AP | 1:1000 |
| Anti-CCNE1 | Proteintech | 11554-1-AP | 1:1500 |
| Anti-CDC25B | Proteintech | 10644-1-AP | 1:500 |
| Anti-TGFB1 | Proteintech | 21898-1-AP | 1:3000 |
| Anti-DTNBP1 | Proteintech | 11132-1-AP | 1:1500 |
| Anti-Tublin | Proteintech | 66031-1-Ig | 1:20000 |
| HRP-Goat Anti-Rabbit IgG(H+L) | Proteintech | SA00001-17 | 1:10000 |
