# Supplementary material for: Objective to identify and verify the regulatory mechanism of DTNBP1 as a prognostic marker for hepatocellular carcinoma
Source: Sci Rep. 2022 Jan 7;12:211. doi: 10.1038/s41598-021-04055-4 (PMC8742032; doi:10.1038/s41598-021-04055-4)
Supplement: Supplementary file 5 — Supplementary Table 3. [file 41598_2021_4055_MOESM5_ESM.docx]

**Supplemental table 3. Core genes contributing to the leading-edge subset in the Kyoto encyclopedia of genes and genomes (KEGG) pathway analysis**

| **Functional category** | **Core genes** |
| --- | --- |
| Cell cycle | YWHAZ, ANAPC7, RBX1, PTTG1, CDC25B, MCM3, CDC20, CCNE1, CCND3, CDKN1C, CDKN2D, YWHAB, TGFB1, HDAC1, HDAC2, CCNB1, YWHAE, LK1, MCM7, YWHAH, CDK7, CDK4, CDC6, CCNB2, RBL1, ANAPC11, CDK1, E2F2, CDC25A, ANAPC4, SKP1, ANAPC5, CDC45, E2F3, ORC1, CHEK1, ORC6, E2F1, CCND2, CDC25C, ZBTB17, MCM6, E2F4, MCM2, CDC16, SFN, MCM4, MCM5, PCNA, PKMYT1, BUB1, TP53, TGFB3, CDC7, SMC1B, DBF4, CHEK2, MAD1L1, BUB3, YWHAQ |
| DNA replication | POLE4, RFC4, RFC2, MCM3, RNASEH2C, PRIM2, RNASEH2B, RNASEH2A, MCM7, POLA2, FEN1, POLD1, LIG1, SSBP1, RPA2, POLD3, MCM6, MCM2, MCM4, MCM5, PCNA, RNASEH1, RPA1 |
