# Supplementary material for: Objective to identify and verify the regulatory mechanism of DTNBP1 as a prognostic marker for hepatocellular carcinoma
Source: Sci Rep. 2022 Jan 7;12:211. doi: 10.1038/s41598-021-04055-4 (PMC8742032; doi:10.1038/s41598-021-04055-4)
Supplement: Supplementary file 6 — Supplementary Information 6. [file 41598_2021_4055_MOESM6_ESM.docx]

**Figure 6B Hep3B**


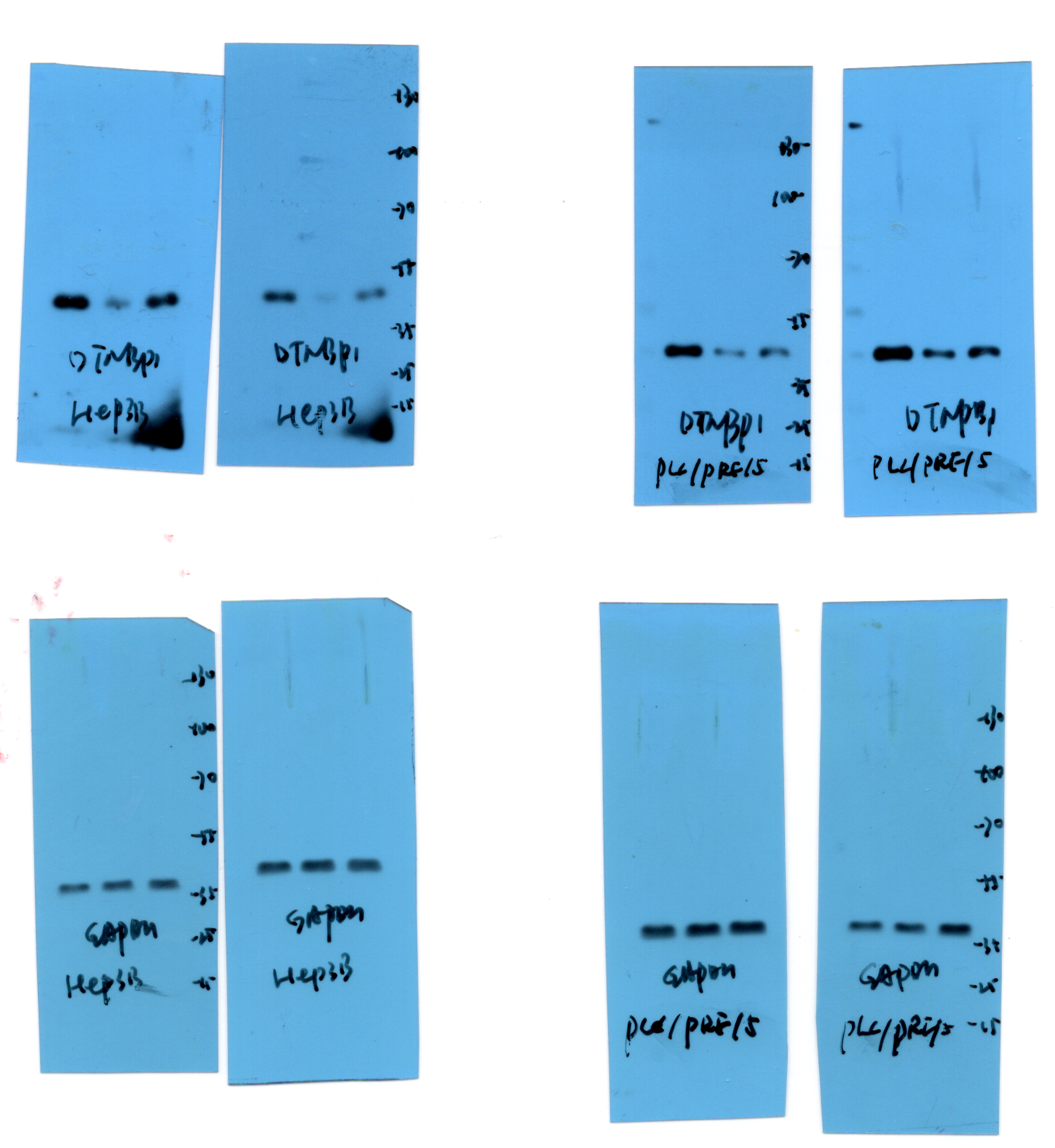


**GAPDH**

**DTNBP1**

**Figure 6B PLC-PRF-5**


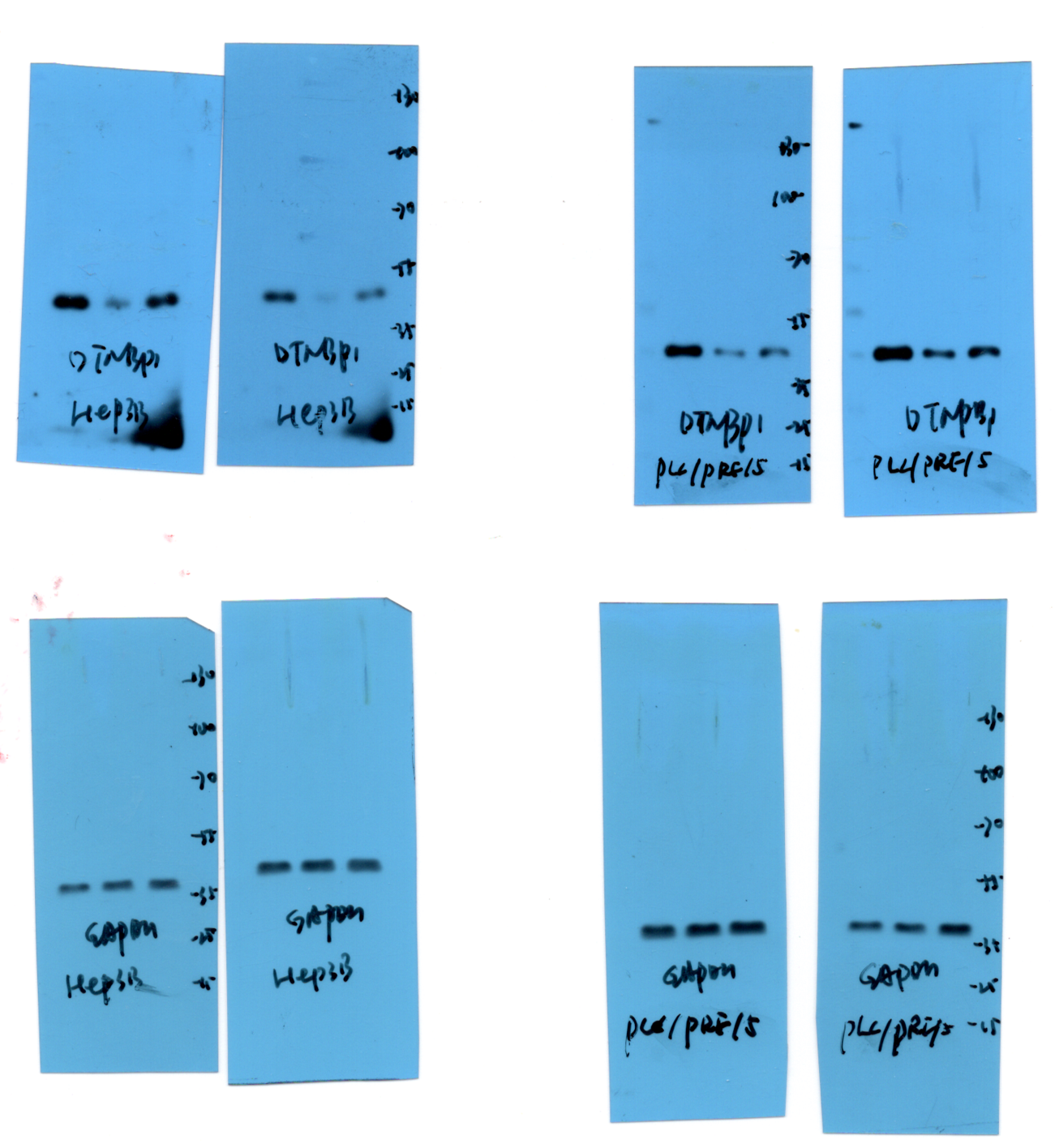


**DTNBP1**

**GAPDH**

**Figure 9C Hep3B**


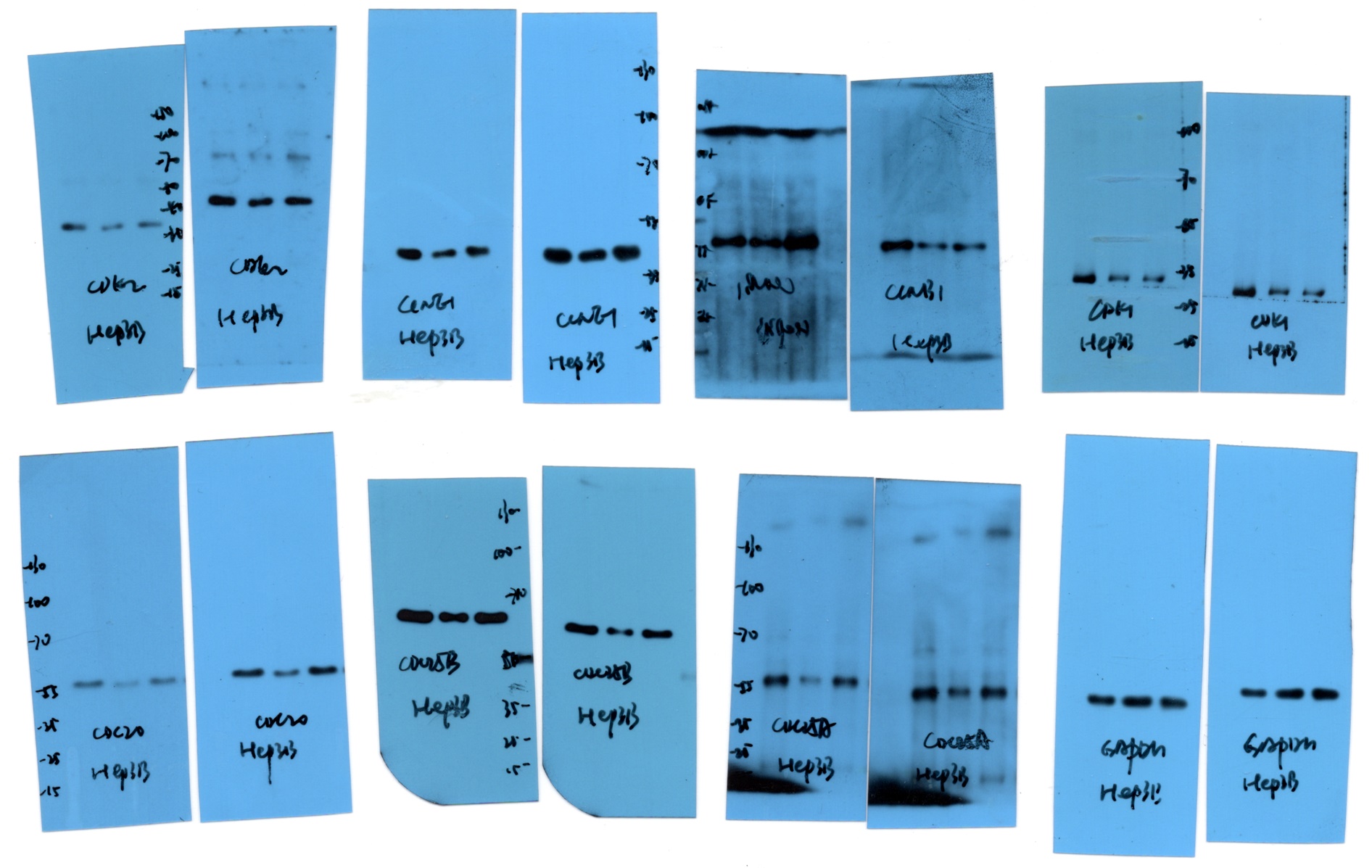


**GAPDH**

**CDC25A**

**CDC25B**

**CDC20**

**CDK1**

**CCNB1**

**CCNE1**

**CDK2**

**Figure 9C** **PLC-PRF-5**


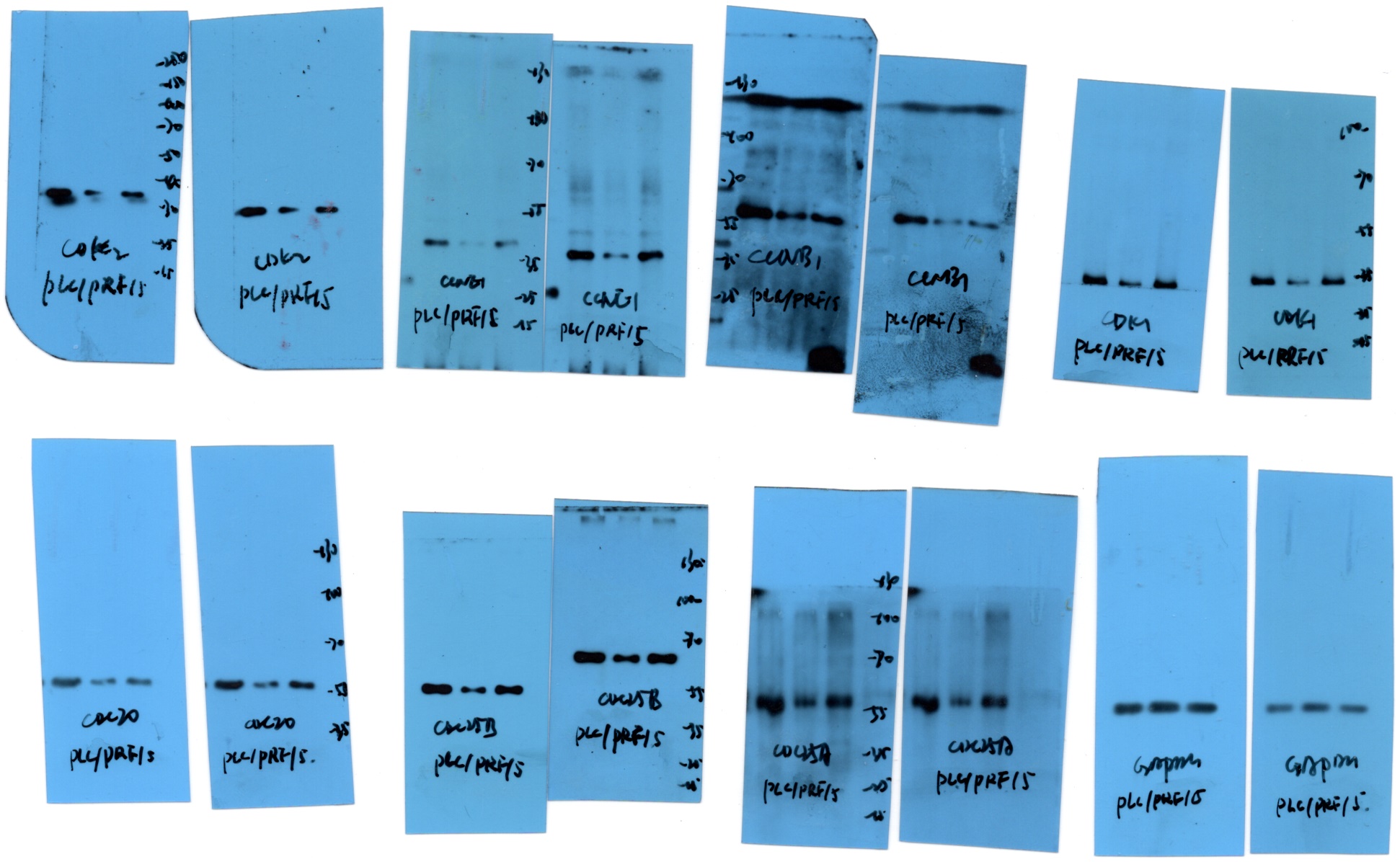


**GAPDH**

**CDC25A**

**CDC25B**

**CDC20**

**CDK1**

**CCNB1**

**CCNE1**

**CDK2**

**Figure 10A Huh7**


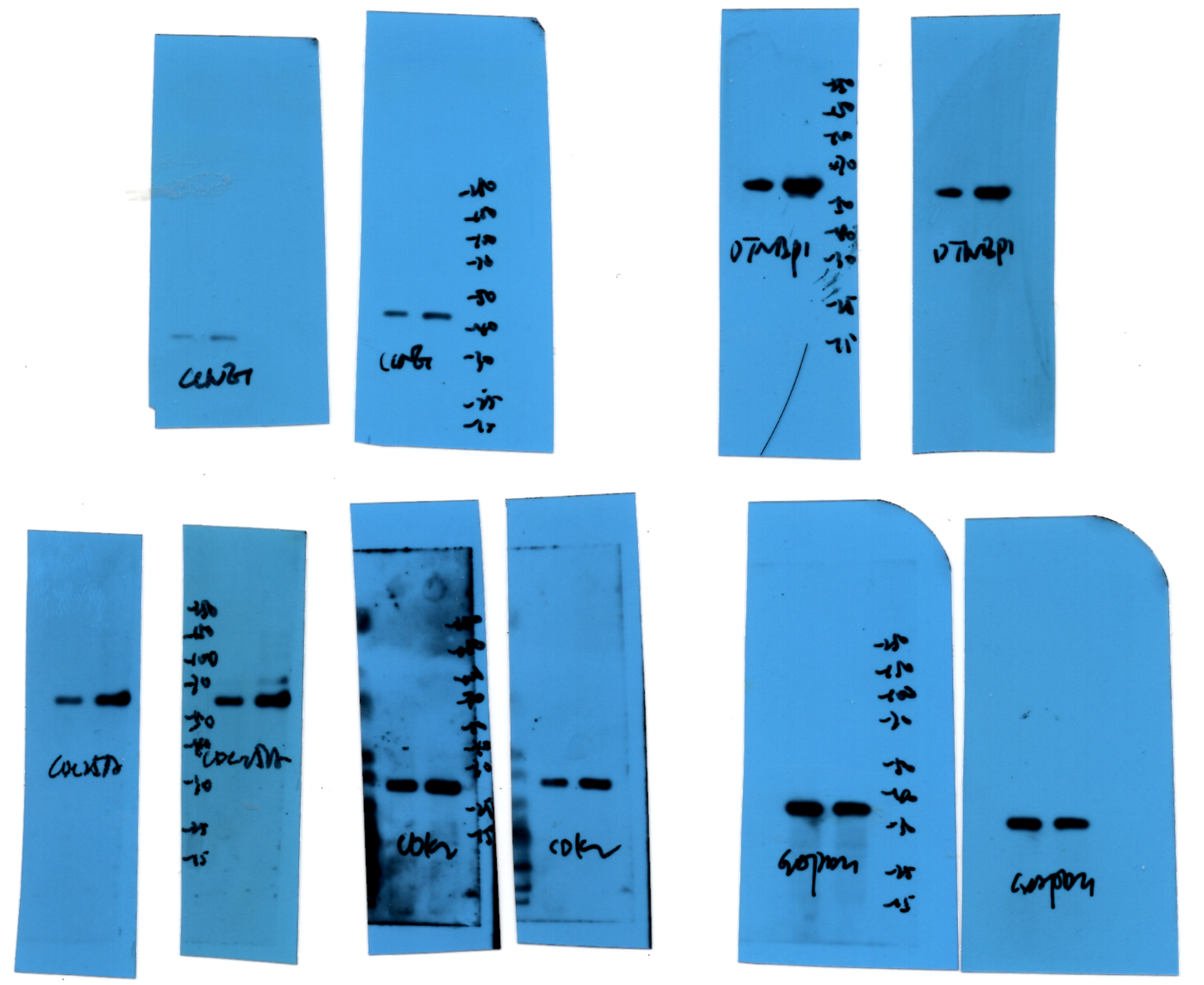


**GAPDH**

**CDK2**

**CDC25A**

**DTNBP1**

**CCNB1**

**Figure S2B**


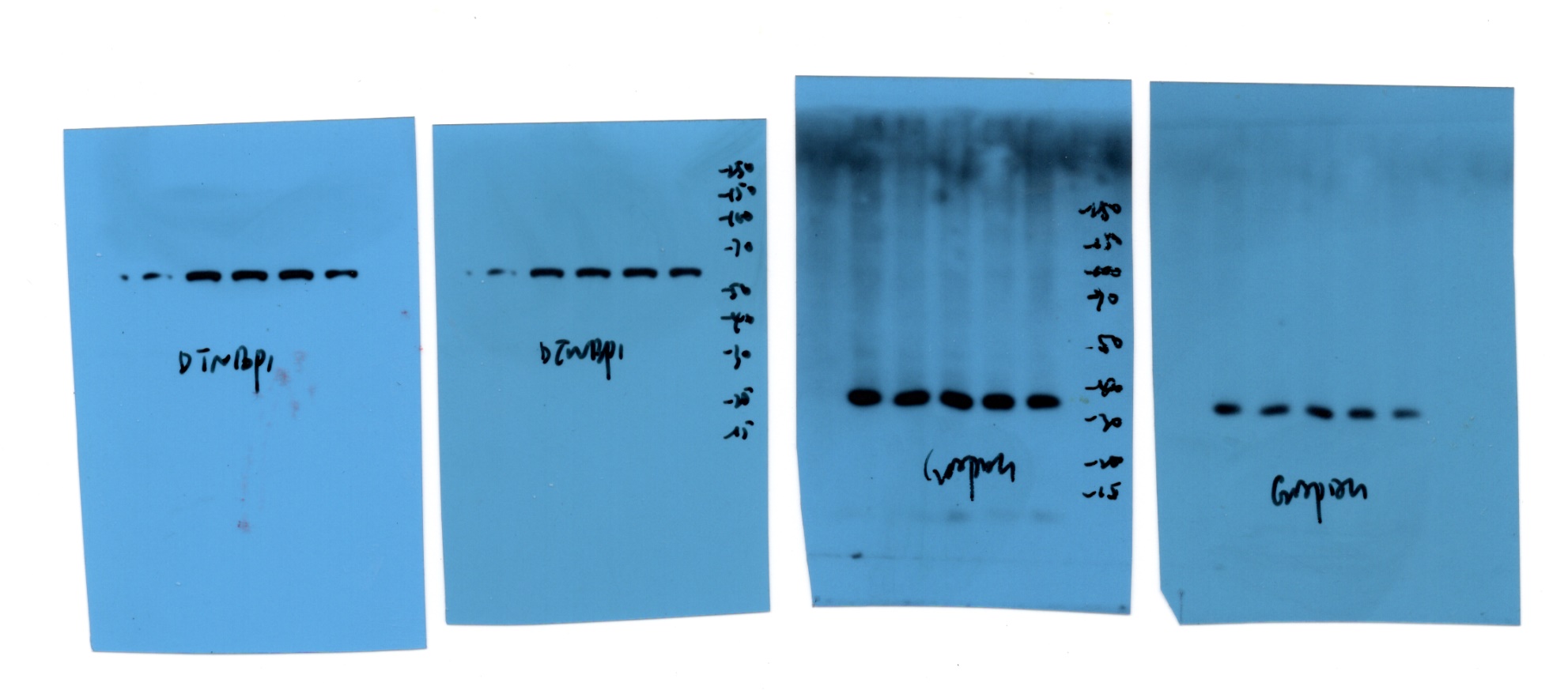


**GAPDH**

**DTNBP1**
